# SAGA–CORE subunit Spt7 is required for correct Ubp8 localization, chromatin association and deubiquitinase activity

**DOI:** 10.1186/s13072-020-00367-3

**Published:** 2020-10-28

**Authors:** Carme Nuño-Cabanes, Varinia García-Molinero, Manuel Martín-Expósito, María-Eugenia Gas, Paula Oliete-Calvo, Encar García-Oliver, María de la Iglesia-Vayá, Susana Rodríguez-Navarro

**Affiliations:** 1 Gene Expression and RNA Metabolism Laboratory, Instituto de Biomedicina de Valencia, Consejo Superior de Investigaciones Científicas (CSIC), C/Jaume Roig 11, 46010 Valencia, Spain; 2 Gene Expression and RNA Metabolism Laboratory, Centro de Investigación Príncipe Felipe (CIPF), C/E. Primo Yúfera 3, 46012 Valencia, Spain; 3 Brain Connectivity Lab. Joint Unit FISABIO & Centro de Investigación Príncipe Felipe (CIPF), C/E. Primo Yúfera 3, 46012 Valencia, Spain

**Keywords:** SAGA, Histone deubiquitination, Spt7, Transcription, Yeast

## Abstract

**Background:**

Histone H2B deubiquitination is performed by numerous deubiquitinases in eukaryotic cells including Ubp8, the catalytic subunit of the tetrameric deubiquitination module (DUBm: Ubp8; Sus1; Sgf11; Sgf73) of the Spt-Ada-Gcn5 acetyltransferase (SAGA). Ubp8 is linked to the rest of SAGA through Sgf73 and is activated by the adaptors Sus1 and Sgf11. It is unknown if DUBm/Ubp8 might also work in a SAGA-independent manner.

**Results:**

Here we report that a tetrameric DUBm is assembled independently of the SAGA–CORE components *SPT7*, *ADA1* and *SPT20*. In the absence of SPT7, i.e., independent of the SAGA complex, Ubp8 and Sus1 are poorly recruited to SAGA-dependent genes and to chromatin. Notably, cells lacking Spt7 or Ada1, but not Spt20, show lower levels of nuclear Ubp8 than wild-type cells, suggesting a possible role for SAGA–CORE subunits in Ubp8 localization. Last, deletion of *SPT7* leads to defects in Ubp8 deubiquitinase activity in in vivo and in vitro assays.

**Conclusions:**

Collectively, our studies show that the DUBm tetrameric structure can form without a complete intact SAGA–CORE complex and that it includes full-length Sgf73. However, subunits of this SAGA–CORE influence DUBm association with chromatin, its localization and its activity.

## Background

The conserved transcription coactivator Spt-Ada-Gcn5 acetyltransferase (SAGA) complex acts during different phases of gene expression [1–5]. Many studies of the molecular architecture of the SAGA complex have shown that flexibility and modularity are key features of its multifunctionality. In yeast, the first low-resolution 3D model of SAGA indicated the existence of five domains and shed light on the functional organization of the complex [6]. Different SAGA domains are involved in its interaction with activators, in TBP binding and in histone modifications [7, 8]. For the latter task, SAGA uses two enzymes within separate modules: the histone acetyltransferase (HAT) Gcn5 in the HAT module (HATm) [9], and the deubiquitinase (DUB) Ubp8 that is part of the DUB module (DUBm) composed of Ubp8, Sus1, Sgf11 and Sgf73 [10–12]. Three other studies have provided insights into SAGA architecture, flexibility and subunit arrangement [13–15]. In the first study, single-particle electron microscopy showed that SAGA contains five modular domains that are arranged in two lobes: Lobe A (containing the biggest SAGA subunit, Tra1), and Lobe B [13]. In that study, the DUBm was mapped within the complex as close to Gcn5 and Spt8 (a yeast-specific subunit that is not present in mammals or in the yeast SAGA-like complex (SLIK) [16]. The second study investigated the subunit interaction network within SAGA by combining chemical cross-linking and mass spectrometry [14]. In this new model, a SAGA hub is occupied by the TFIID-like TAF complex. Moreover, interactions were found between the two enzymatic modules (DUBm and HATm) which, together with the first study, suggest that both enzymatic activities are coordinated within the complex. The third study concluded that SAGA is flexible and is composed of a “core” module (CORE) that supports peripheral catalytic modules [15]. Those authors used several strategies to analyze SAGA and proposed a spatial arrangement of SAGA subunits that explains its flexible adoption of three distinct conformations. Recently, two new studies reporting cryo-EM structures significantly increased our knowledge of SAGA stoichiometry and protein–protein interactions [17, 18]. They show that most of the proteins from the modules previously known as SPT and TAF form a structural CORE complex involving Spt7, Spt3, Spt20, Ada1, Taf5, Taf6, Taf9, Taf10, Taf12 and some residues of Sgf73 and Ada3 [19]. This structural CORE module contains proteins with histone-binding histone fold (HF) motifs that are part of a SAGA octamer-like structure. This octamer-like structure is assembled by components with HF motifs that bind histones, and they contribute to this structure by forming heterodimers with other CORE subunits. One of the most important outcomes of these structural studies is the visualization of the intricate assembly of this SAGA–CORE module. Both reported structures allow visualization of an octamer-like structure at the edge of the SAGA central lobe. The octamer-like structure is composed of paired HF proteins linked to each other in the order: an initial HF Taf6–Taf9 pair, followed by the Taf12–Ada1 pair, then the Taf10–Spt7 pair, and then a final Spt3 HF pair [19]. In addition, those studies further revealed a close connection between the HAT and the DUB activities of SAGA on chromatin.

The tetrameric DUBm has also been investigated using structural approaches [20–23]. Analysis of the DUBm bound to ubiquitinated nucleosomes showed that, while the Sgf11 of DUBm mediates contacts between the DUBm complex and the H2A/H2B dimer, Ubp8 additionally bridges H2B and ubiquitin [23]. Moreover, novel cryo-EM structures demonstrated that the DUBm and HATm complexes contact each other prior to binding to the nucleosome and can adopt different strategies for anchoring to the CORE complex and for retaining flexibility [17, 18]. It was further shown that the DUBm is displaced from the CORE in the chromatin bound state, which highlights the in vivo relevance of these structural changes [18]. Regarding DUB function, all DUBm subunits are essential for maximum deubiquitinase activity in vivo and in vitro [24]. Moreover, the Sus1 adaptor protein component of DUBm also forms part of the nuclear pore-associated complex required for mRNA export (the TREX-2 complex) [25–27].

Although there is some evidence suggesting that DUBm might function independently of SAGA, little is known regarding its mechanism of action under this condition. Here, we demonstrate that a complete four-subunit DUBm, including the region of Sgf73 that is incorporated in the SAGA–CORE, can be assembled independently of specific SAGA–CORE subunits. In this context, Ubp8 is mostly dissociated from chromatin and its nuclear localization is decreased. We found similarities in the effects of deletion of either of the two CORE subunits Spt7 and Ada1 on Ubp8 cellular localization, in contrast to deletion of Spt20. Our results suggest an increase in global levels of monoubiquitinated H2B in the absence of *SPT7*, which is consistent with a lower deubiquitinase activity of Ubp8 in vitro. We describe new biochemical and functional data of a SAGA-independent DUBm that highlight the key role of Spt7 and Ada1 in Ubp8 nuclear localization, and a potential role for Spt7 in modulating Ubp8 deubiquitinase activity.

## Results

### A tetrameric DUBm containing full-length Sgf73 can form without a complete intact SAGA–CORE complex

The DUBm can be separated from the rest of SAGA by increasing salt concentration or by deleting SAGA subunits such as *SGF73* or *SPT20* [25–28]. Traditionally, Spt20, together with Spt7 and Ada1, were considered as the three CORE structural subunits of SAGA [29]. However, very recent structural studies of the SAGA complex showed that Spt20 can also contact the SAGA Lobe A (interacting with Tra1). In contrast, Spt7 and Ada1 have crucial roles in the octamer-like structure in the SAGA Lobe B that is close to Sgf73 [17, 18]. In the light of these new structural findings, we determined if Spt7 or Ada1 might play a role in DUBm assembly by investigating the ability of the DUBm to assemble in the absence of these subunits of the SAGA–CORE. Using Sus1-TAP the DUBm was purified from wild-type or mutant cells lacking *SPT7*, or lacking *ADA1* and/or *SPT20*. In all cases, only the DUBm and TREX-2 subunits were identified in mass spectrometric analysis of the proteins co-purifying with Sus1-TAP (Table 1 and Additional file 1: Table S1); no other SAGA components co-purified with Sus1-TAP. In contrast, purification of Sus1-TAP from other SAGA mutants such as *sgf29*Δ (mutation of a HATm subunit), did not affect Sus1 co-purification with the remaining SAGA subunits (Additional file 1: Table S1). We also found that Ubp8-TAP co-purified with the DUBm components Sus1, Sgf11 and Sgf73, but not with other SAGA subunits in *spt7*Δ cells (Table 1). These findings reinforce the idea that a tetrameric DUBm can assemble in vivo, without the participation of other SAGA components.

**Table 1:**
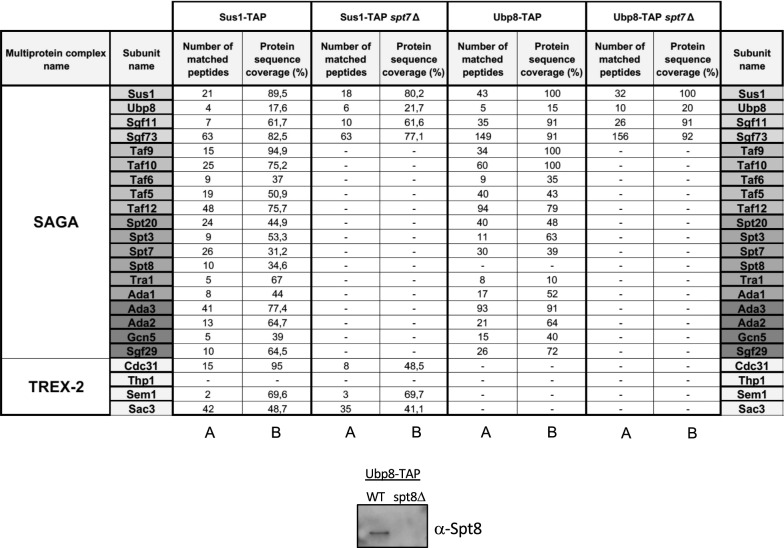
List of proteins identified by LC MS/MS and MASCOT software in purifications of Sus1-TAP and Ubp8-TAP from different mutants of SAGA components

The presence of Spt8 in Upb8-TAP was verified by Western-blotting. (A) Number of matched peptides; (B) protein sequence coverage (%)

Sgf73 functions as a link between the DUBm and the SAGA–CORE through residues 353–437 [18]. Notably, peptides corresponding to all regions of Sgf73, including residues 353–437, were identified in all the Sus1-TAP precipitates from CORE mutants (Table 1 and Additional file 2: Fig. S1) indicating that full-length Sgf73 is present in the DUBm in cells lacking a functional SAGA–CORE. The primary conclusion from these experiments is that a tetrameric DUBm containing full-length Sgf73 is indeed assembled independently of Spt7 or Ada1.

### Deletion of the SAGA–CORE subunit *SPT7* weakens DUBm association with genes and chromatin

**Fig. 1:**
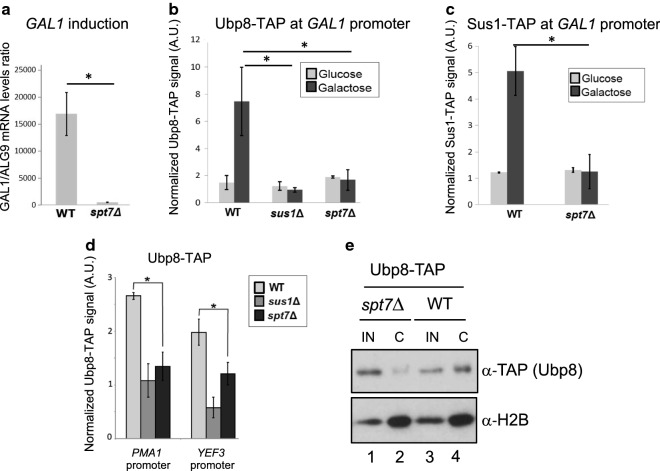
Association of Ubp8 with chromatin depends on Spt7. **a** qPCR analysis of *GAL1* gene expression levels in WT and *spt7*Δ strains. **b**, **c** Level of Ubp8-TAP (**b**) or Sus1-TAP (**c**) associated with the SAGA-regulated gene *GAL1* was monitored using ChIP analysis in WT and *spt7*Δ strains (**b**, **c**) and in the *sus1*Δ strain (**b** only, negative control) under inhibitory (glucose) or activation conditions (galactose). **d** Levels of Ubp8-TAP associated with the *PMA1* and *YEF3* promoters were monitored using ChIP analysis in WT, *sus1*Δ and *spt7*Δ strains. In **b**–**d** promoter occupancy level was calculated as the ratio of the IP sample signal to the input signal. A.U., arbitrary units. Error bars denote the SD of at least three independent experiments in each panel. The ratios normalized with respect to an intergenic region from at least three independent experiments are shown. In **a**–**d** * indicates one-tailed unpaired Student’s t-test *p*-value < 0.05. **e** Inputs (IN) and chromatin-enriched fractions (C) of the Ubp8-TAP strain (WT) and its isogenic mutant *spt7*Δ, were subjected to western blotting to detect Ubp8 (α-TAP, upper panel). Enrichment of chromatin-associated proteins in the C fraction was determined by detection of total histone H2B (α-H2B, lower panel). Cropped blots are shown for clarity. Full-length blots are presented in Additional file 2: Fig. S3. All samples were run in the same gel

When DUBm is part of the SAGA complex, nucleosomal H2Bub1 is its main substrate, and a prominent role for Sgf11 in targeting Ubp8 to its substrate has been reported [20, 23]. To investigate whether the DUBm assembled in *spt7*Δ cells could still access this substrate, we performed chromatin-IP (ChIP) analyses of the association of Ubp8 and Sus1 with the promoter of the SAGA-regulated gene *GAL1* in wild-type and *spt7*Δ cells, since Spt7 has a prominent role in *GAL1* activation (Fig. 1a). We found that both Ubp8-TAP (Fig. 1b) and Sus1-TAP (Fig. 1c) were inefficiently recruited to the *GAL1* gene promoter in *spt7*Δ cells compared to wild type (WT), which is consistent with a role for Spt7 in recruitment of these proteins to the promoter. Additionally, there was also a significant Spt7 requirement for the association of Ubp8 with chromatin at the promoters of other genes such as *PMA1* and *YEF3* (Fig. 1d). Moreover, Ubp8 was readily detected in purified chromatin-enriched fractions from WT cells (Fig. 1e, lane 4) but not from *spt7*Δ cells (Fig. 1e, lane 2), suggesting that Spt7 is required for global association of Ubp8 with chromatin. We concluded that, whereas the DUBm purified from *spt7*Δ cells is highly stable, its association with chromatin is weakened compared to in WT cells.

### The SAGA–CORE complex influences the deubiquitinase activity of Ubp8 in vitro

**Fig. 2:**
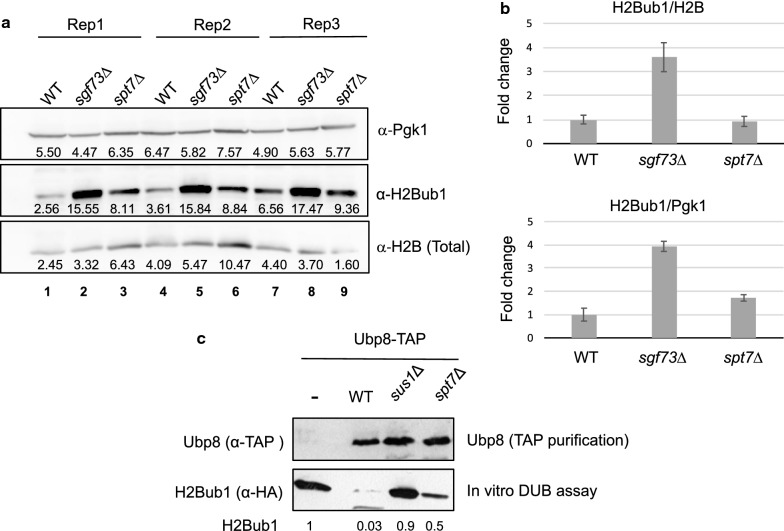
Participation of Spt7 in Ubp8-dependent H2B deubiquitination. **a** Whole cell extracts were obtained from WT, *sgf73*Δ and *spt7*Δ cells, and levels of H2Bub1 were monitored by western blotting using an anti-α-H2Bub1 antibody. Levels of Pgk1 and total H2B were analyzed as loading controls. Cropped blots are shown for clarity. Full-length blots are presented in Additional file 2: Fig. S4 Rep1/2/3 shown in the figure correspond to three independent replicas. **b** Quantification of panel **a** showing the ratio of H2Bub1/H2B of three independent experiments. Error bars represent standard deviation. **c** Ubp8-TAP was purified via TAP from wild-type (WT, lane 2), *sus1*Δ (lane 3) and *spt7*Δ (lane 4) strains. The purified Ubp8-TAPs were then incubated with purified histone H2B (containing HA-ubiquitin modified H2B and unmodified H2B) and in vitro H2Bub1 deubiquitination was assayed. Purified histone H2B alone was incubated with buffer and used as a negative control (lane 1(−)). H2B monoubiquitin levels are indicated at the bottom relative to the level in lane 1(−), which was given an arbitrary value of 1. The values are representative of at least three independent experiments. Cropped blots are shown for clarity. Full-length blots are presented in Additional file 2: Fig. S5

Using *spt7*Δ cells as a proxy for a non-intact SAGA–CORE complex, we concluded that a full DUBm formed independently of the CORE in vivo is inefficiently targeted to chromatin. However, since all four of its subunits are present in this DUBm, its enzymatic activity might be retained. In this regard, contradictory results in vivo have been reported over the last few years. Baptista et al. [30] showed increased levels of H2Bub1 in the absence of Spt7, suggesting that Ubp8 activity is reduced in vivo under this condition [30]. In contrast, Donczew et al. [31] recently reported that total H2Bub1 levels are reduced in *spt7*Δ cells, suggesting that Ubp8 could be hyperactivated or that H2B ubiquitination is not fully functional in this mutant. In our hands, only a minor increase in H2Bub1 levels was observed by western blotting upon *SPT7* deletion (Fig. 2a, lanes 3, 6 and 9, α-H2Bub1), whereas deletion of *SGF73* significantly increased H2Bub1 levels versus WT (Fig. 2a, lanes 2, 5 and 8). Thus, our results are in better agreement with those of Baptista et al. [30], than with those of Donczew et al. [31]. However, a slight increase in the total H2B level versus WT was also observed in the absence of *SPT7* (Fig. 2a, lanes 3, 6 and 9, α-H2B total). Therefore, although a trend for augmentation of the H2Bub1 level with respect to the WT was reproducible in the *spt7*Δ mutant, the difference in H2Bub1 levels relative to total levels of H2B was negligible (Fig. 2b).

We further studied the role of Spt7 in DUBm mediation of the H2Bub1 level by the alternative approach of an in vitro H2Bub1 deubiquitination assay. In this assay, in contrast to other in vitro experiments performed with the reconstituted module, the DUBm was purified via Ubp8-TAP from growing cells lacking Spt7 or Sus1, or from the WT strain (Fig. 2c, α-TAP). These DUBm were then incubated with an equal amount of the substrate H2Bub1 (FLAG-tagged H2B/HA-ubiquitin). Quantification of the amount of H2Bub1 remaining after incubation, by using western blotting with an anti-HA antibody, was used as a readout of DUBm activity. While Ubp8 purified from the WT strain decreased H2Bub1 levels from a value of 1 [arbitrarily assigned to the control incubation without Ubp8 (−)] to 0.03 (Fig. 2c, α-HA), Ubp8 purified in the absence of *SUS1* did not significantly reduce the levels of H2Bub1 (0.9 vs. 1, Fig. 2c, α-HA, and [32]). Notably, a 50% reduction in the H2Bub1 signal was observed when Ubp8 was purified from *spt7*Δ yeast cells (0.5 vs. 1, Fig. 2c, α-HA). These results suggested that the DUBm purified from growing *spt7*Δ cells does not retain maximal Upb8 deubiquitinase activity.

### Spt7 and Ada1 modulate nuclear localization of Ubp8

**Fig. 3:**
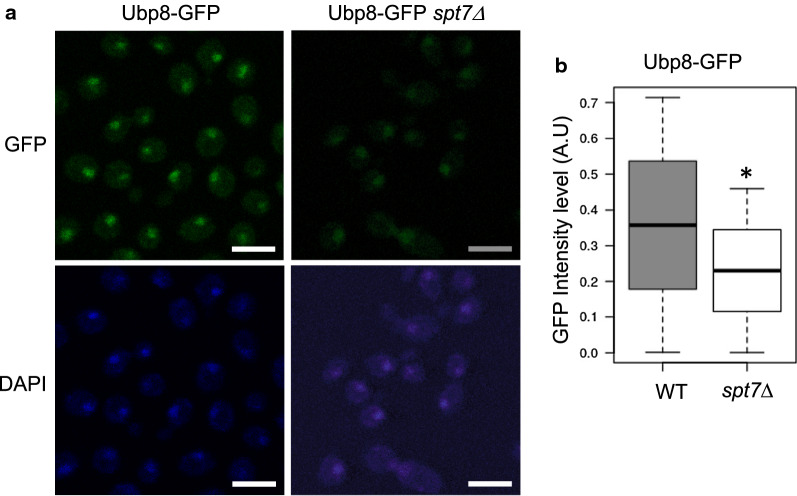
Spt7 is a key factor for Ubp8 cellular localization. **a** Localization of Ubp8 tagged with GFP in WT and *spt7*Δ cells was monitored using fluorescence microscopy. Nuclei were stained with DAPI (DNA). Images were cropped from full sized frames of ~ 250–500 cells and scale bars are set to 5.0 µm. **b** Boxplot indicating GFP nuclear intensity for Ubp8-GFP (WT) and Ubp8-GFP*spt7*Δ strains obtained from more than 100 cells

The above experiments show that the association of Upb8 with chromatin and the deubiquitination of H2Bub1 are modulated by the SAGA–CORE. Since both of these functions take place in the nucleus it is possible that the SAGA–CORE influences nuclear localization of the DUBm. To investigate if the absence of Spt7 might affect the localization of Ubp8, we tagged Ubp8 with GFP in wild-type and *spt7*Δ strains and then analyzed GFP-Ubp8 cellular localization using fluorescence microscopy. Figure 3 shows that deletion of *SPT7* significantly (*p* < 0.001) reduced the nuclear signal of Ubp8-GFP (Fig. 3a, b). We confirmed that this effect was not due to a lower expression of the Ubp8-GFP fusion protein in *spt7*Δ versus the WT cells by western blot analysis of Ubp8-GFP (α-GFP) expression in total protein extracts from two independent replicates from each of three experiments, using Pgk1 as a loading control (Additional file 2: Fig. S2).

**Fig. 4:**
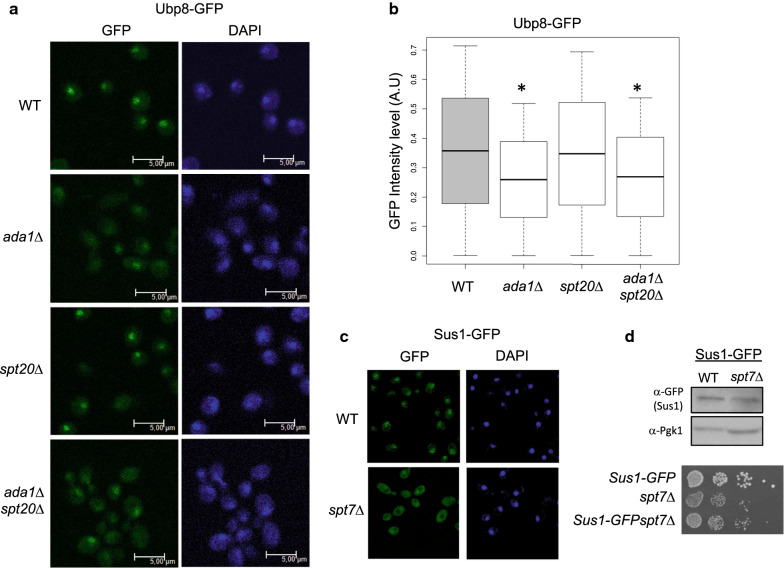
Ada1 is a key factor in Ubp8 cellular localization. **a** Localization of Ubp8 tagged with GFP in WT, *ada1*Δ, *spt20*Δ and *ada1*Δ*spt20*Δ cells was monitored using fluorescence microscopy. Nuclei were stained with DAPI (DNA). Images were cropped from full sized frames of ~ 250–500 cells and scale bars are set to 5.0 µm. **b** Boxplot indicating GFP nuclear intensity for Ubp8-GFP WT, Ubp8-GFP*ada1*Δ, Ubp8-GFP*spt20*Δ and Ubp8-GFP*ada1*Δ*spt20*Δ strains obtained from more than 100 cells in each case. **c** Localization of the Sus1 protein tagged with GFP in WT and *spt7*Δ was monitored using fluorescence microscopy. Nuclei were stained with DAPI (DNA). **d** Upper panel: levels of Sus1 protein tagged with GFP in WT and *spt7*Δ mutant cells were analyzed by western blotting of whole-cell extracts using an anti-α-GFP antibody. Levels of Pgk1 protein were monitored as the loading control. Lower panel: serial dilutions of WT and mutant *spt7*Δ cells, expressing or not expressing Sus1-GFP-tagged proteins as indicated, were grown on YPD plates. Plates were incubated for 48 h at 30 °C

Previous data suggested that Spt20, Ada1 and Spt7 are the main SAGA–CORE subunits, each contributing differently to SAGA assembly, stability and function [29]. Spt7 appears to have the strongest effect on SAGA assembly, whereas in *ada1*Δ and *spt20*Δ cells some SAGA subunits still interact with Spt7. More recently, as mentioned above, it was shown that Ada1 and Spt7 are located in the SAGA–CORE (Lobe B) and are part of the histone octameric-like structure (Ada1–Taf12 and Taf10–Spt7 HF pairs), whereas Spt20 contacts with the SAGA Lobe A [17, 18]. Therefore, to determine the potential role of these SAGA–CORE subunits in DUBm localization, we compared the localization of GPF-tagged Ubp8 in *ada1*Δ and *spt7*Δ cells with that in *spt20*Δ cells. Similar to *SPT7* loss, the absence of *ADA1* led to a reduction in the Ubp8-GFP nuclear signal (Fig. 4a, b). In contrast, deletion of *SPT20* had no effect on Ubp8 localization, since no significant differences in its nuclear signal were observed in *spt20*Δ cells compared to the WT (Fig. 4a, b). The double mutant *ada1*Δ*spt20*Δ also displayed an *ada1*Δ phenotype, suggesting that, although Spt20 is known to affect SAGA stability and conformation, these effects do not modulate nuclear accumulation of Ubp8.

Since the absence of Spt7 also affected Sus1 recruitment to the *GAL1* gene (Fig. 1c) and its association with SAGA (Table 1), we also analyzed how deletion of *SPT7* might affect the subcellular localization of Sus1. As shown in Fig. 4c, the localization of Sus1-GFP to the nucleus was decreased in *spt7*Δ cells. This phenotype was not due to Sus1 protein instability as indicated by western blotting of Sus1-GFP expression in WT and *spt7*Δ cell extracts (Fig. 4d, upper panel). Furthermore, it was also not caused by a synergistic growth defect produced by tagging Sus1 in the *spt7*Δ mutant since the growth of Sus1-GFP *spt7*Δ was comparable to that of *spt7*Δ (Fig. 4d, lower panel). We concluded that Spt7 deletion similarly affects Sus1-GFP and Ubp8-GFP nuclear localization.

## Discussion

The SAGA co-activator is a paradigm of a multisubunit complex with distinct functions (see recent reviews in [19, 33–37]). Although identified more than 20 years ago, there remain many open questions about its function and modularity. In the light of new high-resolution structural studies [17, 18] some of the existing results can now be better interpreted and integrated into a more comprehensive model. In this study, we present a combination of molecular and genetic experiments to address specific questions regarding DUBm in relation to the SAGA–CORE. The main conclusions from these experiments are: (i) the tetrameric DUBm can form in the absence of a complete intact SAGA–CORE complex; (ii) this DUBm contains full-length Sgf73, including the amino acids that reside inside the CORE; (iii) the SAGA–CORE influences DUBm association with chromatin and its deubiquitinating activity; and iv) specific SAGA–CORE subunits are required for normal DUBm nuclear localization.

Previous studies by others and us showed that the DUBm can be detached from SAGA and suggested that a free DUBm can exist independently of the formation of the SAGA complex. That DUBm is not always associated with SAGA has been shown in different studies in yeast, Arabidopsis and humans (see a recent revision in [38]). For instance in Arabidopsis, DUBm association with SAGA is regulated by light, highlighting its dynamic nature in this organism [39]. However, the precise role of SAGA–CORE subunits in the SAGA–DUBm interaction in vivo is not fully understood. Our present result that Sgf73 is incorporated into the DUBm independently of the other SAGA components is very interesting in the light of new structural data showing that the central Sgf73 residues 353–437 are part of the SAGA–CORE, thereby connecting the CORE to the DUBm [18]. This finding indicates that interaction of these residues with the SAGA–CORE is not necessary for DUBm assembly as a tetramer. This result could imply that a fully (or at least partially) assembled SAGA–CORE may facilitate contacts between Sgf73 and the CORE and thereby DUBm association with SAGA.

**Fig. 5:**
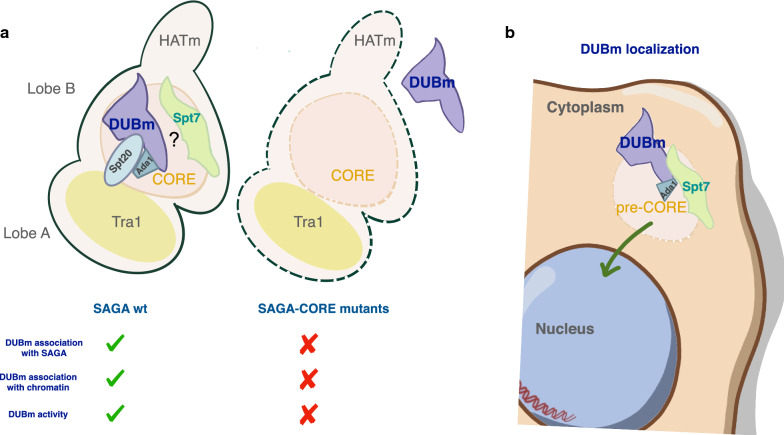
Intact SAGA–CORE complex influences DUBm functions and localization. **a** In SAGA–CORE mutants *spt7*Δ, *ada1*Δ, *spt20*Δ SAGA is not assembled (dash lines) and the DUBm is free. This DUBm contains full length Sgf73, including the amino acids that reside inside the CORE. The SAGA–CORE is required for DUBm association with chromatin and its deubiquitinating activity. **b** Spt7 and Ada1, but not Spt20 are required for normal DUBm nuclear localization. A possible explanation is that assembly of pre-CORE particles facilitates the nuclear transport of the DUBm. SAGA model based on [49]

Our experiments showing differences between CORE components in terms of their impact on Ubp8 nuclear localization are more interesting in the light of recent SAGA structural studies. The three SAGA–CORE subunits used in this study were selected since they were traditionally considered the “key CORE” components. This concept has been updated based on all of the new structural studies. The ability of both Spt7 and Ada1, and the inability of Spt20, to modulate Ubp8 localization is consistent with the recent new knowledge regarding the protein–protein interactions between Spt7–Taf10 and Ada1–Taf12 as part of the SAGA–CORE histone octamer-like fold that is involved in binding of the Ubp8 substrate, whereas Spt20 contacts with Tra1 in a different SAGA lobe (Lobe A). In this regard it is notable that a physical interaction between Ada1 and Spt7 in an Spt7–TAP purification from *spt20*Δ cells was reported years ago [29]. These results regarding the effects of deletion of Spt7, Ada1 and/or Spt20 on Ubp8-GFP localization are intriguing since our biochemical experiments demonstrated that the DUBm forms independently of SAGA in all three mutants**.** These findings leave open the possibility of the existence of weak Ubp8/SAGA–CORE or other unknown interactions that would help to explain the observed differences. However, the fact that Ubp8 localization is not affected by deletion of *SPT20* challenges a simple model in which defective integrity of SAGA leads to problems in DUBm transport. A more complicated network of physical interactions between the SAGA subunits must therefore be invoked to explain our results. Spt7 has been reported to be involved in regulating the levels of Spt20 and Ada1, suggesting that Spt7 levels control the amount of SAGA present in vivo [29]. We speculate that the observed differences in Ubp8 localization between *spt7*Δ, *ada1*Δ and *spt20*Δ mutants might be due to the formation of partial Spt7 or Ada1-containing SAGA complexes that could be assembled in the absence of Spt20 [29] and might contribute to Ubp8 localization (Fig. 5). Several studies emphasize the importance of the different SAGA subunits for the assembly and nuclear import of the complex. For instance, co-translational assembly of Ada2 and Spt20, which is essential for their nuclear localization, depends on Not5, a subunit of the Ccr4-Not complex [40]. Moreover, the pseudokinase domain of the SAGA subunit Tra1 has also been shown to be involved in SAGA’s import into the nucleus [41]. Further research is required to clarify how the DUBm is transported into the nucleus in yeast cells and why Ubp8 nuclear accumulation is differentially affected by deletion of Spt7, Ada1 and Spt20. Previous, structural studies showed that the DUBm maps close to Gcn5 and Spt7 [13]. This observation is compatible with our results regarding Ubp8- and Sus1–Spt7 functional interactions and highlights the specific role of Spt7 in the DUBm–SAGA interaction. In this respect it is interesting that loss of Sgf73 favors the cleavage of the C-terminal ends of the Spt7 subunit and the loss of the Spt8 subunit from SAGA [13]. This could be indicative of a close positioning of DUBm and Spt7. Nevertheless, other studies have suggested that Spt7 does not directly contact DUBm subunits [14, 15], and, more recently, cryo-EM studies did not detect direct interactions between DUBm subunits and SAGA other than for the DUBm subunit Sgf73 [17, 18].

Our experiments also confirmed that DUBm association with chromatin is modulated by the SAGA–CORE. Part of Sgf73 resides in the SAGA–CORE thereby facilitating binding of the Ubp8 nucleosomal histone substrate. The formation of heterodimeric pairs of CORE subunits (HF pairs) is responsible for SAGA assembly at promoters with the correct orientation for histone binding. In the absence of *SPT7*, the pair of Spt7–Taf10 would not form, which would prevent SAGA association with the histone octamer leading to poor access of Ubp8 to nucleosomal H2Bub1. The newly reported cryo-EM structure of the SAGA nucleosome complex shows that the DUBm is displaced from the SAGA–CORE in a chromatin-bound state [18, 42]. The flexibility of the SAGA complex and the presence of different interconnected submodules allow SAGA to modify different histones substrates in a regulated manner. It has been proposed that the DUBm would deubiquitinate promoter-bound nucleosomes around or downstream of the transcription start site. Deletion of the CORE component Spt7 would result in a drastic conformational change in SAGA that could have an impact on Ubp8 activity on the histones in these specific nucleosomes, but may not affect Ubp8 activity at other locations. Such a model could explain the differences in H2Bub1 levels observed in the different studies and might explain why the lower levels of nuclear Ubp8 observed in this study in the absence of Spt7 are sufficient to deubiquitinate most of the total H2Bub1. We consider that the above-described discrepancies between laboratories regarding the participation of Spt7 in modulating H2Bub1 levels could also be due to the use of specific strain backgrounds, antibodies, growth and histone purification conditions that make it difficult to quantify the role of Spt7 in maintaining total H2Bub1 levels in vivo. Such levels could be very sensitive to experimental conditions. Nevertheless, to really examine this issue would likely require a totally different strategy, or some means of circumventing some of the problems of how the experiments are perform and quantified.

In sum, we favor a model in which Spt7 plays a prominent role in DUBm association with SAGA and in its activation at the positions where its specific substrates that are SAGA-dependent are located. Further mechanistic studies are required to fully understand the participation of each SAGA subunit in its precise in vivo functions at its specific genomic locations and to identify the alternative targets of the DUBm that are totally independent of SAGA.

## Methods

### Yeast strains, recombinant DNA and microbiological techniques

The yeast strains used in this study are listed in Additional file 1: Table S2. Microbiological techniques were carried out essentially as previously described 29. Chromosomal integration of a C-terminally tagged cassette into the TAP or GFP-tagged strains was performed as previously described [43, 44]. For gene disruption, the indicated gene was deleted using a PCR product amplified from either the KanMX4 plasmid pRS400 or the HIS3 plasmid pFA6a. All deletions and genomically tagged strains were confirmed by PCR analysis and/or western blot analysis.

Growth assays were carried out by growing cells at 30 °C in YPD to an OD_600_ of 0.3–0.4. Subsequently, tenfold serial dilutions of an equal number of cells were made and drops of these dilutions were spotted onto YPD plates. Growth was recorded after 48 h of incubation at 30 °C.

### Preparation of yeast cell extracts, TAP purifications and western blot analysis

To prepare yeast cell extracts, yeast cultures were grown to an OD_600_ of 0.5–0.8 in YPD medium. Total proteins were extracted using the trichloroacetic acid (TCA) method. All tandem affinity purifications (TAPs) were performed as previously described [45]. Briefly, TAP-fusion proteins and their associated proteins were recovered from cell extracts by affinity selection on IgG Sepharose beads. After bead washing, the Tobacco Etch Virus (TEV) protease was added to release the bound material. The eluate was incubated with calmodulin-coated beads in the presence of calcium. After washing, the bound material was released with EGTA. This enriched fraction was called the calmodulin eluate. To analyze the TAP-purified protein complexes, TCA-precipitation, LysC/trypsin digestion and multidimensional protein identification technology (MudPIT) mass spectrometry analyses were performed as described previously [46]. Following electrophoresis and western blotting, membranes were probed with specific antibodies: α-PGK1 was used as a loading control (Invitrogen), α-HA (Roche), α-TAP (Thermo Fisher), α-H2B total (Active Motif), α-H2Bub1 (Cell Signaling), α-Spt8 (Santa Cruz) and α-GFP (Roche). Quantification of the western blot bands was performed by densitometry using ImageJ software and subsequent normalization using the ratio between the protein to study and loading control protein.

### Chromatin immunoprecipitation and chromatin-enriched fractions

Chromatin immunoprecipitation (ChIP) was performed as previously described [32]. In brief, early log-phase cultures (100 mL), grown in YP medium containing 2% raffinose, were separated into two aliquots and either glucose or galactose was added to one aliquot to a final concentration of 2%. Thirty min after the addition of each carbon source, cultures were cross-linked with 1% of formaldehyde solution (Sigma) for 20 min at room temperature with intermittent shaking. After quenching with 125 mM glycine, cells were collected by centrifugation and washed four times with 25 mL cold Tris–saline buffer (150 mM NaCl and 20 mM Tris–HCl at pH 7.5). Cells were broken in 300 µL of lysis buffer [50 mM HEPES–KOH at pH 7.5, 140 mM NaCl, 1 mM EDTA, 10% glycerol, 0.5% Tergitol-type NP-40 (NP-40), 1 mM phenylmethylsulfonyl fluoride (PMSF) and protease inhibitors (Complete, Roche)] plus glass beads. Cell extracts were sonicated in a Bioruptor sonicator (Diagenode) for 30 min of 30 s on/30 s off cycles to yield chromatin fragments with an average size of 300 bp. An aliquot (10 µL) of extract was reserved as the input and the rest was used for immunoprecipitation with magnetic beads (Dynabeads^®^) coated with monoclonal anti-mouse IgG antibodies. Immunoprecipitations were conducted for 2 h at 4 °C, and the immune complexes were then washed twice with 1 mL of lysis buffer, twice with 1 mL of lysis buffer supplemented with 360 mM NaCl, twice with 1 mL wash buffer (10 mM Tris–HCl at pH 8.0, 250 mM LiCl, 0.5% NP-40, 5 mg/mL of nadeoxycholol and 1 mM EDTA) and once with 1× TE. Samples were eluted at 65 °C for 15 min with 100 µL of elution buffer (50 mM Tris–HCl at pH 8, 10 mM EDTA and 1% SDS). Inputs and immunoprecipitation (IP) samples were incubated overnight at 65 °C to reverse the cross-link. Samples were then treated with proteinase K (Ambion), at 100 mg/250 mL of chromatin for 2 h. Afterwards, DNA was extracted twice with phenol:chloroform:isoamyl alcohol (25:24:1) and once with chloroform:isoamyl alcohol (24:1), and was then ethanol precipitated and resuspended in 40 µL of 1× TE. DNA was used as a template in the qPCR reaction using specific primers for GAL1, PMA1 and YEF3 promoters (Additional file 1: Table S3).

Chromatin-enriched fractions (ChEFs) were obtained from 50 mL of cells with an OD_600_ of 0.5. Cells were collected by centrifugation, were washed with water and were broken by resuspending in 200 µL of buffer 1 (HEPES 20 mM at pH 8, KCl 60 mM, NaCl 15 mM, MgCl_2_ 10 mM, CaCl2 1 mM, butyric acid 10 mM, triton X-100 0.8%, sucrose 0.25 M, spermidine 2.5 mM and spermine 0.5 mM) plus 200 µL of glass beads and vortexing for 4 min at 4 °C. All of the following steps were conducted at 4 °C. Lysate was then centrifuged for 5 min at 500×*g* and the supernatant was recovered and re-centrifuged once more for 5 min at 500×*g*. The new supernatant was recovered in a new tube and a 20-µL sample from this extract was used as INPUT (IN). The rest of the extract was centrifuged at 20,000×*g* for 20 min and the pellet was resuspended in 200 µL of buffer 1 and centrifuged at 20,000×*g* for 20 min. After discarding the supernatant, the pellet was resuspended in 200 µL of buffer 2 (HEPES pH 7.6 20 mM, NaCl 450 mM, MgCl2 7.5 mM, EDTA 20 mM, glycerol 10%, NP-40 1%, sucrose 0.5 M, urea 2 M, DTT 1 mM and PMFS 0.125 mM) and centrifuged at 20,000×*g* for 20 min. The supernatant was discarded and the pellet was again resuspended in buffer 2 and centrifuged at 20,000×*g* for 20 min. The supernatant was discarded and the pellet, which was used as CHROMATIN FRACTION (C), was resuspended in 20 µL of Laemmli buffer 1× to be run on a gel.

### Quantitative RT-PCR

Total RNA prepared by hot phenol extraction was treated for 30 min at 30 °C with DNase I RNase-free (Roche) prior to use for cDNA synthesis. Subsequently, cDNA was synthetized in 20 µL reactions containing 50 ng/µL of DNase I treated RNA, 250 ng of random hexamers (Invitrogen), 10 units/µL of SuperScript III Reverse Transcriptase (Invitrogen), 1× First Strand Buffer, 10 mM DTT, and 0.5 mM dNTPs, following the manufacturer’s instructions. Quantitative real-time PCR was then performed in a LightCycle 480 Thermal Cycler (Roche) using the SYBR^®^ Premix Ex Taq™ kit (Takara) for fluorescent labeling. For each analysis primer pair, a negative control was included. The primers set used in this study is provided in Additional file 1: Table S3. A primer pair for *ALG9* was used as a reference gene. Data and errors bars represent the average and standard deviation of three independent biological samples.

### In vitro deubiquitination assay

The FLAG-tagged H2B substrate (including H2B ubiquitinated with HA-ubiquitin and unmodified H2B) was obtained by purifying N-terminally Flag-tagged histone H2B using an M2 agarose slurry (Sigma A2220) from cells that lack genomic *HTB1* gene and contain two plasmids, one in which histone H2B is tagged with FLAG tag (pZS145 HTA1-Flag-HTB1-CEN-HIS3) and other in which Ubiquitin protein is tagged with HA epitope (GAPDH-3HAUB14::URA3). The purified substrate was split into equal aliquots each containing 500 ng of FLAG-tagged H2B. Aliquots were incubated with Ubp8-TAP purified complexes at room temperature for 30 min in deubiquitination (DUB) buffer (100 mM Tris–HCl at pH 8.0, 1 mM EDTA, 1 mM DTT, 5% glycerol, 1 mM PMSF and 1% protease inhibitor). One aliquot was subjected to a mock in vitro deubiquitination reaction lacking Ubp8-TAP. Reactions were stopped by adding one volume of 2× Laemmli sample buffer containing 50 mM DTT. Samples were separated on a 15% SDS-PAGE gel, transferred to a nitrocellulose membrane and subjected to western blot analysis with an anti-HA antibody (Roche) that was used to detect HA-tagged ubiquitin.

### Fluorescence microscopic analysis of GFP localization

Yeast cultures (10 mL) that were grown to an OD_600_ of 0.3–0.6 were pelleted and were then fixed by incubating them in methanol for 10 min on ice while vortexing every 2 min. Fixed cells were subsequently centrifuged and washed once with 1× PBS (137 mM NaCl, 2.7 mM KCl, 10 mM Na2HPO4, 1.8 mM KH2PO4). Cells were resuspended in 30–50 µL of 1× PBS and then 10 µL was placed on glass microscope slides. Samples were observed under a Leica TCS-SP2-AOBS confocal microscope.

### Quantification of GFP fluorescence microscopic data

GFP fluorescence microscopic data were quantified using IPython notebooks. Find full description in Additional file 2: Fig. S8. The main library used for the pipeline implementation was Scikit-image [47]. The GPF and DAPI images were first converted to grayscale images and were represented by a NumPy array [48] to construct a histogram of pixel values from the GFP nuclear intensity values. Several components of the Scikit-image library were combined into an image processing workflow as follows: (i) binarization—images were converted to their binarized version to discriminate GFP nuclear intensity from noise background. We employed the filter threshold Otsu algorithm, and we further removed the artifacts connected to the image border; (ii) segmentation—to count the cells, a segmentation of the DAPI cell nuclei was performed; (iii) statistical analysis—R package software [50] was used to generate the statistical analysis. To determine significant values between different experimental groups, the mean data were compared using one-way analysis of variance (ANOVA). Tukey's multiple comparisons test was also used. Values of *p < 0.001 were considered significant.

## Supplementary information

**Additional file 1: Table S1.** List of proteins identified by mass spectrometry in Sus1-TAP precipitates from Sus1-TAP ada1Δ, Sus1-TAP spt20Δ and Sus1-TAP sgf29Δ. **Table S2.** List of strains used in this study. **Table S3.** Oligonucleotides used in this study.

**Additional file 2: Figure S1. **Sgf73 peptides detected by LC MS/MS and MASCOT in Sus1-TAP precipitates from different SAGA mutants. The middle region of Sgf73, which was recently found to contact SAGA-CORE subunits [17] is highlighted in blue. Some of the Sgf73 peptides found by LC MS/MS are highlighted in red. **Figure S2. **Levels of Ubp8 protein tagged with GFP in WT and *spt7*Δ mutant cells were analysed in three different experiments (1, 2 and 3) with two replicates of Ubp8-GFP*spt7*Δ in each (a and b) by western blotting of whole-cell extracts using an anti-GFP antibody. Levels of Pgk1 protein were also monitored and used as the loading control. Cropped blots are shown for clarity. Full-length blots are presented in Figure S6. **Figure S3.** Full-length blot that was cropped for Fig. 1e. **Figure S4. **Full-length blot that was cropped for Fig. 4a. **Figure S5.** Full-length blot that was cropped for Fig. 4c (upper and lower panels). **Figure S6. **Full-length blots that were cropped for Figure S2 (upper panel). **Figure S7.** Different full microscope images used in Figs. 2 and 3. **Figure S8.** Detailed GFP Fluorescence microscopic quantification using IPython notebooks. To determine significant values between different experimental groups, the mean data were compared using one-way analysis of variance (ANOVA). Tukey's multiple comparisons test was also used. Values of *p < 0.001 were considered significant.

## Data Availability

All data generated or analyzed during this study are included in this published article and its Additional files.

## Abbreviations

DUBm: Deubiquitination module
SAGA: Spt-Ada-Gcn5 acetyltransferase
TBP: TATA box binding protein
HAT: Histone acetyltransferase
SLIK: SAGA-like complex
TFIID: Transcription factor II D
HF: Histone fold
TAP: Tandem affinity purification
ChIP: Chromatin immunoprecipitation
WT: Wild type
GFP: Green fluorescent protein
cryo-EM: Cryo-electron microscopy
TREX-2: Transcription and export complex 2
TCA: Trichloroacetic acid
ChEF: Chromatin-enriched fraction

## References

[1] Koutelou E, Hirsch CL, Dent SYR (2010). Multiple faces of the SAGA complex. Curr Opin Cell Biol.

[2] Weake VM, Workman JL (2012). SAGA function in tissue-specific gene expression. Trends Cell Biol.

[3] Samara NL, Wolberger C (2011). A new chapter in the transcription SAGA. Curr Opin Struct Biol.

[4] Spedale G, Timmers HTM, Pijnappel WWMP (2012). ATAC-king the complexity of SAGA during evolution. Genes Dev.

[5] Rodríguez-Navarro S (2009). Insights into SAGA function during gene expression. EMBO Rep.

[6] Wu PYJ, Ruhlmann C, Winston F, Schultz P (2004). Molecular architecture of the *S. cerevisiae *SAGA complex. Mol Cell.

[7] Timmers HTM, Tora L (2005). SAGA unveiled. Trends Biochem Sci.

[8] Pijnappel WWMP, Timmers HTM (2008). Dubbing SAGA unveils new epigenetic crosstalk. Mol Cell.

[9] Grant PA, Duggan L, Côté J, Roberts SM, Brownell JE, Candau R (1997). Yeast Gcn5 functions in two multisubunit complexes to acetylate nucleosomal histones: characterization of an Ada complex and the SAGA (Spt/Ada) complex. Genes Dev.

[10] Ingvarsdottir K, Krogan NJ, Emre NCT, Wyce A, Thompson NJ, Emili A (2005). H2B ubiquitin protease Ubp8 and Sgf11 constitute a discrete functional module within the *Saccharomyces cerevisiae* SAGA complex. Mol Cell Biol.

[11] Lee KK, Florens L, Swanson SK, Washburn MP, Workman JL (2005). The deubiquitylation activity of Ubp8 is dependent upon Sgf11 and its association with the SAGA complex. Mol Cell Biol.

[12] Köhler A, Pascual-García P, Llopis A, Zapater M, Posas F, Hurt E (2006). The mRNA export factor Sus1 is involved in Spt/Ada/Gcn5 acetyltransferase-mediated H2B deubiquitinylation through its interaction with Ubp8 and Sgf11. Mol Biol Cell.

[13] Durand A, Bonnet J, Fournier M, Chavant V, Schultz P (2014). Mapping the deubiquitination module within the SAGA complex. Structure.

[14] Han Y, Luo J, Ranish J, Hahn S (2014). Architecture of the *Saccharomyces cerevisiae* SAGA transcription coactivator complex. EMBO J.

[15] Setiaputra D, Ross JD, Lu S, Cheng DT, Dong M-Q, Yip CK (2015). Conformational flexibility and subunit arrangement of the modular yeast Spt-Ada-Gcn5 acetyltransferase complex. J Biol Chem.

[16] Pray-Grant MG, Schieltz D, McMahon SJ, Wood JM, Kennedy EL, Cook RG (2002). The novel SLIK histone acetyltransferase complex functions in the yeast retrograde response pathway. Mol Cell Biol.

[17] Papai G, Frechard A, Kolesnikova O, Crucifix C, Schultz P, Ben-Shem A (2020). Structure of SAGA and mechanism of TBP deposition on gene promoters. Nature.

[18] Wang H, Dienemann C, Stützer A, Urlaub H, Cheung ACM, Cramer P (2020). Structure of the transcription coactivator SAGA. Nature.

[19] Helmlinger D, Papai G, Devys D, Tora L (2020). What do the structures of GCN5-containing complexes teach us about their function?. Biochim Biophys Acta Gene Regul Mech.

[20] Samara NL, Datta AB, Berndsen CE, Zhang X, Yao T, Cohen RE (2010). Structural insights into the assembly and function of the SAGA deubiquitinating module. Science.

[21] Köhler A, Zimmerman E, Schneider M, Hurt E, Zheng N (2010). Structural basis for assembly and activation of the heterotetrameric SAGA histone H2B deubiquitinase module. Cell.

[22] Yan M, Wolberger C (2015). Uncovering the role of Sgf73 in maintaining SAGA deubiquitinating module structure and activity. J Mol Biol.

[23] Morgan MT, Haj-Yahya M, Ringel AE, Bandi P, Brik A, Wolberger C (2016). Structural basis for histone H2B deubiquitination by the SAGA DUB module. Science.

[24] Workman JL (2016). CHROMATIN. It takes teamwork to modify chromatin. Science.

[25] Pascual-García P, Govind CK, Queralt E, Cuenca-Bono B, Llopis A, Chavez S (2008). Sus1 is recruited to coding regions and functions during transcription elongation in association with SAGA and TREX2. Genes Dev.

[26] Köhler A, Schneider M, Cabal GG, Nehrbass U, Hurt E (2008). Yeast Ataxin-7 links histone deubiquitination with gene gating and mRNA export. Nat Cell Biol.

[27] Lee KK, Swanson SK, Florens L, Washburn MP, Workman JL (2009). Yeast Sgf73/Ataxin-7 serves to anchor the deubiquitination module into both SAGA and Slik(SALSA) HAT complexes. Epigenet Chromatin.

[28] Lee KK, Sardiu ME, Swanson SK, Gilmore JM, Torok M, Grant PA (2011). Combinatorial depletion analysis to assemble the network architecture of the SAGA and ADA chromatin remodeling complexes. Mol Syst Biol.

[29] Wu PYJ, Winston F (2002). Analysis of Spt7 function in the *Saccharomyces cerevisiae* SAGA coactivator complex. Mol Cell Biol.

[30] Baptista T, Grünberg S, Minoungou N, Koster MJE, Timmers HTM, Hahn S (2017). SAGA is a general cofactor for RNA polymerase II transcription. Mol Cell.

[31] Donczew R, Warfield L, Pacheco D, Erijman A, Hahn S (2020). Two roles for the yeast transcription coactivator SAGA and a set of genes redundantly regulated by TFIID and SAGA. Elife.

[32] García-Oliver E, Pascual-García P, García-Molinero V, Lenstra TL, Holstege FCP, Rodríguez-Navarro S (2013). A novel role for Sem1 and TREX-2 in transcription involves their impact on recruitment and H2B deubiquitylation activity of SAGA. Nucleic Acids Res.

[33] Nuño-Cabanes C, Rodríguez-Navarro S (2020). The promiscuity of the SAGA complex subunits: multifunctional or moonlighting proteins?. Biochim Biophys Acta Gene Regul Mech.

[34] Strahl BD, Briggs SD (2020). The SAGA continues: the rise of cis- and trans-histone crosstalk pathways. Biochim Biophys Acta Gene Regul Mech.

[35] Cheon Y, Kim H, Park K, Kim M, Lee D (2020). Dynamic modules of the coactivator SAGA in eukaryotic transcription. Exp Mol Med.

[36] Grasser KD, Rubio V, Barneche F (2020). Multifaceted activities of the plant SAGA complex. Biochim Biophys Acta Gene Regul Mech.

[37] Espinola Lopez JM, Tan S (2020). The Ada2/Ada3/Gcn5/Sgf29 histone acetyltransferase module. Biochim Biophys Acta Gene Regul Mech.

[38] Goswami R, Parra DVC, Allan S, Costanzo K, Morales-Sosa P, Mohan RD (2020). Function and regulation of the Spt-Ada-Gcn5-acetyltransferase (SAGA) deubiquitinase module. Biochim Biophys Acta Gene Regul Mech.

[39] Nassrallah A, Rougée M, Bourbousse C, Drevensek S, Fonseca S, Iniesto E (2018). DET1-mediated degradation of a SAGA-like deubiquitination module controls H2Bub homeostasis. Elife.

[40] Kassem S, Villanyi Z, Collart MA (2017). Not5-dependent co-translational assembly of Ada2 and Spt20 is essential for functional integrity of SAGA. Nucleic Acids Res.

[41] Berg MD, Genereaux J, Karagiannis J, Brandl CJ (2018). The pseudokinase domain of *Saccharomyces cerevisiae* Tra1 is required for nuclear localization and incorporation into the SAGA and NuA4 complexes. G3 (Bethesda).

[42] Soffers JHM, Li X, Saraf A, Seidel CW, Florens L, Washburn MP (2019). Characterization of a metazoan ADA acetyltransferase complex. Nucleic Acids Res.

[43] Longtine MS, McKenzie A, Demarini DJ, Shah NG, Wach A, Brachat A (1998). Additional modules for versatile and economical PCR-based gene deletion and modification in *Saccharomyces cerevisiae*. Yeast.

[44] Gavin A-C, Bösche M, Krause R, Grandi P, Marzioch M, Bauer A (2002). Functional organization of the yeast proteome by systematic analysis of protein complexes. Nature.

[45] Rodríguez-Navarro S, Fischer T, Luo M-J, Antúnez O, Brettschneider S, Lechner J (2004). Sus1, a functional component of the SAGA histone acetylase complex and the nuclear pore-associated mRNA export machinery. Cell.

[46] Pamblanco M, Oliete-Calvo P, García-Oliver E, Luz Valero M, Sanchez del Pino MM, Rodríguez-Navarro S (2014). Unveiling novel interactions of histone chaperone Asf1 linked to TREX-2 factors Sus1 and Thp1. Nucleus.

[47] van der Walt S, Schönberger JL, Nunez-Iglesias J, Boulogne F, Warner JD, Yager N (2014). scikit-image: image processing in Python. PeerJ.

[48] Van der Walt S, Colbert SC, Varoquaux G (2011). The NumPy array: a structure for efficient numerical computation. CiSE.

[49] Soffers JHM, Popova VV, Workman JLW (2020). SAGA structures provide mechanistic models for gene activation. Trends Biochem Sci.

[50] R Core Team. R: A language and environment for statistical computing. R Foundation for Statistical Computing, Vienna, Austria. https://www.R-project.org/. 2018.

