# Supplementary material for: SAGA–CORE subunit Spt7 is required for correct Ubp8 localization, chromatin association and deubiquitinase activity
Source: Epigenetics Chromatin. 2020 Oct 28;13:46. doi: 10.1186/s13072-020-00367-3 (PMC7594455; doi:10.1186/s13072-020-00367-3)
Supplement: Supplementary file 1 — Additional file 1: Table S1. List of proteins identified by mass spectrometry in Sus1-TAP precipitates from Sus1-TAP ada1Δ, Sus1-TAP spt20Δ and Sus1-TAP sgf29Δ. Table S2. List of strains used in this study. Table S3. Oligonucleotides used in this study. [file 13072_2020_367_MOESM1_ESM.docx]

**Additional file 1**

**Table S1.** List of proteins identified by mass spectrometry in Sus1-TAP precipitates from Sus1-TAP ada1Δ, Sus1-TAP spt20Δ and Sus1-TAP sgf29Δ

|  |  | **Sus1-TAP *ada1*Δ** | **Sus1-TAP *spt20*Δ** | **Sus1-TAP *sgf29*Δ** |
| --- | --- | --- | --- | --- |
| **Multiprotein complex name** | **Subunit name** | **Number of matched peptides** | **Number of matched peptides** | **Number of matched peptides** |
| **SAGA** | **Sus1** | 12 | 7 | 6 |
|  | **Ubp8** | 22 | 15 | 12 |
|  | **Sgf11** | 3 | 3 | 2 |
|  | **Sgf73** | 19 | 4 | 16 |
|  | **Taf9** | - | - | 1 |
|  | **Taf10** | - | - | 1 |
|  | **Taf6** | - | - | 11 |
|  | **Taf5** | - | - | 16 |
|  | **Taf12** | - | - | 15 |
|  | **Spt20** | - | - | 15 |
|  | **Spt3** | - | - | 5 |
|  | **Spt7** | - | - | 18 |
|  | **Spt8** | - | - | 10 |
|  | **Tra1** | - | - | 41 |
|  | **Ada1** | - | - | 8 |
|  | **Ada3** | - | - | 11 |
|  | **Ada2** | - | - | 7 |
|  | **Gcn5** | - | - | 6 |
|  | **Sgf29** | - | - | - |
| **TREX-2** | **Cdc31** | 8 | 1 | 7 |
|  | **Thp1** | 39 | 41 | 4 |
|  | **Sem1** | - | - | - |
|  | **Sac3** | 44 | 15 | 58 |

**Table S2.** List of strains used in this study

| **Yeast Strain** | **Genotype** | **Reference** |
| --- | --- | --- |
| BY4741 | *Mat a, leu2-Δ0, his3-Δ1, met15-Δ0, ura3-Δ0* | Euroscarf |
| *spt7*∆ | *Mat a, leu2-∆0, his3-∆1, met15-∆0, ura3-∆0 spt7::KANMX4* | Euroscarf |
| SUS1-TAP | *Mat a, leu2-Δ0, his3-Δ1, met15-Δ0, SUS1-TAP::URA3* | This study |
| SUS1-TAP *spt7*∆ | *Mat a, leu2-Δ0, his3-Δ1, met15-Δ0, SUS1-TAP::URA3 spt7::KanMX4* | This study |
| SUS1-TAP *spt20*∆ | *Mat a, leu2-Δ0, his3-Δ1, met15-Δ0, SUS1-TAP::URA3 spt20::KanMX4* | This study |
| SUS1-TAP *ada1*∆ | *Mat a, leu2-Δ0, his3-Δ1, met15-Δ0, SUS1-TAP::URA3 ada1::KanMX4* | This study |
| SUS1-TAP *sgf29*∆ | *Mat a, leu2-Δ0, his3-Δ1, met15-Δ0, SUS1-TAP::URA3 sgf29::KanMX4* | This study |
| UBP8-TAP | *Mat a, leu2-Δ0, his3-Δ1, met15-Δ0, ura3-Δ0, UBP8-TAP::URA3* | (García-Oliver et al., 2013) |
| UBP8-TAP *sus1*∆ | *Mat a, leu2-Δ0, his3-Δ1, met15-Δ0, ura3-Δ0, UBP8-TAP::URA3, sus1::KanMX4* | This study |
| UBP8-TAP *spt7*∆ | *Mat a, leu2-Δ0, his3-Δ1, met15-Δ0, ura3-Δ0, UBP8-TAP::URA3, spt7::KanMX4* | This study |
| UBP8-GFP | *Mat a, leu2-Δ0, his3-Δ1, met15-Δ0, ura3-Δ0 UBP8-GFP::HIS3* | This study |
| UBP8-GFP *ada1*∆ | *Mat a, leu2-Δ0, his3-Δ1, met15-Δ0, ura3-Δ0 UBP8-GFP::HIS3, ada1::KanMX4* | This study |
| UBP8-GFP sus*1*∆ | *Mat a, leu2-Δ0, his3-Δ1, met15-Δ0, ura3-Δ0 UBP8-GFP::HIS3, sus1::KanMX4* | This study |
| UBP8-GFP *spt7*∆ | *Mat a, leu2-Δ0, his3-Δ1, met15-Δ0, ura3-Δ0 UBP8-GFP::HIS3, spt7::KanMX4* | This study |
| UBP8-GFP *spt20*∆ | *Mat a, leu2-Δ0, his3-Δ1, met15-Δ0, ura3-Δ0 UBP8-GFP::HIS3, spt20::KanMX4* | This study |
| UBP8-GFP spt20∆*ada1*∆ | *Mat a, leu2-Δ0, his3-Δ1, met15-Δ0, ura3-Δ0 UBP8-GFP::HIS3, ada1::KanMX4 spt20::LEU* | This study |

**Table S3.** Oligonucleotides used in this study

| Primers | Target | Sequence 5’-3’ |
| --- | --- | --- |
| 973 | GAL1 (qPCR) | TTGCTAGATCGCCTGGTAGAGTC |
| 974 | GAL1 (qPCR) | GGCGCAAAGCATATCAAAATC |
| 1318 | PMA1 (qPCR) | CAGCTCATCAGCCAACTCAA |
| 1319 | PMA1 (qPCR) | TCGTCGACACCGTGATTAGA |
| 1326 | YEF3 (qPCR) | CACTGCTGACAACAGACACG |
| 1327 | YEF3 (qPCR) | TTGATACCCTTGGCCAATTC |
| 1671 | ALG9 (qPCR) | CCAAGTTCTTTCCTGCTGCC |
| 1672 | ALG9 (qPCR) | TGATCGGCCACTCTTTACCG |
| 971 | GAL1 (CHIP) | AAAATTGGCAGTAACCTGGCC |
| 972 | GAL1 (CHIP) | *CCCCAGAAATAAGGCTAAAAAACTAA* |
| 1316 | PMA1 (CHIP) | AAAAGGCCAAATATTGTATTATTTTCA |
| 1317 | PMA1 (CHIP) | TTCACTATTGGTGTTATAGGAAAGAAA |
| 1324 | YEF3 (CHIP) | TTTTTCGCTTCCTCGAGTATAA |
| 1325 | YEF3 (CHIP) | GAAAGAGAGCGTAAGAAAAAGAGA |
