# Supplementary material for: SAGA–CORE subunit Spt7 is required for correct Ubp8 localization, chromatin association and deubiquitinase activity
Source: Epigenetics Chromatin. 2020 Oct 28;13:46. doi: 10.1186/s13072-020-00367-3 (PMC7594455; doi:10.1186/s13072-020-00367-3)
Supplement: Supplementary file 2 — Additional file 2: Figure S1. Sgf73 peptides detected by LC MS/MS and MASCOT in Sus1-TAP precipitates from different SAGA mutants. The middle region of Sgf73, which was recently found to contact SAGA-CORE subunits [17] is highlighted in blue. Some of the Sgf73 peptides found by LC MS/MS are highlighted in red. Figure S2. Levels of Ubp8 protein tagged with GFP in WT and spt7Δ mutant cells were analysed in three different experiments (1, 2 and 3) with two replicates of Ubp8-GFPspt7Δ in each (a and b) by western blotting of whole-cell extracts using an anti-GFP antibody. Levels of Pgk1 protein were also monitored and used as the loading control. Cropped blots are shown for clarity. Full-length blots are presented in Figure S6. Figure S3. Full-length blot that was cropped for Fig. 1e. Figure S4. Full-length blot that was cropped for Fig. 4a. Figure S5. Full-length blot that was cropped for Fig. 4c (upper and lower panels). Figure S6. Full-length blots that were cropped for Figure S2 (upper panel). Figure S7. Different full microscope images used in Figs. 2 and 3. Figure S8. Detailed GFP Fluorescence microscopic quantification using IPython notebooks. To determine significant values between different experimental groups, the mean data were compared using one-way analysis of variance (ANOVA). Tukey's multiple comparisons test was also used. Values of *p < 0.001 were considered significant. [file 13072_2020_367_MOESM2_ESM.docx]

**Additional file 2**

**Figure S1.** Sgf73 peptides detected by LC MS/MS and MASCOT in Sus1-TAP precipitates from different SAGA mutants. The middle region of Sgf73, which was recently found to contact SAGA-CORE subunits (Papai et al, Nature 2020) is highlighted in blue. Some of the Sgf73 peptides found by LC MS/MS are highlighted in red.

**Sgf73 in Sus1-TAPspt20Δ**

MRSGDAEIKGIKPKVIEEYSLSQGSGPSNDSWKSLMSSAKDTPLQYDHMNRESLKKYFNPNAQLIEDPLDKPIQYRVCEKCGKPLALTAIVDHLENHCAGASGKSSTDPRDESTRETIRNGVESTGRNNNDDDNSNDNNNDDDDDDDNDD NEDDDDADDDDDNSNGANYKKNDSSFNPLKRSTSMESANTPNMDTKRSKT G**T**PQTFSSSI**K**KQKKVKQRNPTE**K**HLIDFNKQCGVELPEGGYCARSLTCK SHSMGAKRAVSGRSKPYDVLLADYHREHQTKIGAAAE**K**RAKQQELQKLQ**K**

QIQKEQKKHTQQQKQGQRSKQRNVNGGKSAKNGGKSTVHNGNNINEIGHV NLT**PEEETTQVLNGVSR**SFPLPLESTVLSSVR**YRTKYFRMREMFASSFSVKPGYTSPGYGAIHSRVGCLDLDRTTDYKFRVRTPQPI**NHLTNQNLNPKQIQRLQQQRALQAQLLSQQQQQQQQQQQHHSPQAQAQASTQQPTQGMVPNHFPGGATNSSFNANVSSKQIQQQQQQQQHKSQDTGLTPLEIQSQQQKLRQQQLQQQKFEAAASYLANATKLMQESNQDSHLSGTHNNNSSKNGNNNLMTMKA SISSPNTSVNSIQSPPSVNSVNGSGQGVSTGINVSGNNGRIEVGIGNSVNPYNGRIN*

**Sgf73 in Sus1-TAPada1Δ**

MRSGDAEIKGIKPKVIEEYSLSQGSGPSNDSWKSLMSSAKDTPLQYDHMNRESLKKYFNPNAQLIEDPLDKPIQYRVCEKCGKPLALTAIVDHLENHCAGASGKSSTDPRDESTRETIRNGVESTGRNNNDDDNSNDNNNDDDDDDDNDD NEDDDDADDDDDNSNGANYKKNDSSFNPLKRSTSMESANTPNMDTKRSKT GTPQTFSSSIKKQKKVKQRNPTEKHLIDFNKQCGVELPEGGYCARSLTCK SHSMGAKRAVSGRSKPYDVLLADYHREHQTKIGAAAEKRAKQQELQKLQK

QIQKEQKKHTQQQKQGQRSKQRNVNGGKSAKNGGKSTVHNGNNINEIGHV NLT**PEEETTQVLNGVSRSFPLPLESTVLSSVRYRTKYFRMREMFASSFSVKPGYTSPGYGAIHSRVGCLDLDRTTDYKFRVRTPQPI**NHLTNQNLNPKQIQRLQQQRALQAQLLSQQQQQQQQQQQHHSPQAQAQASTQQPTQGMVPNHFPGGATNSSFNANVSSKQIQQQQQQQQHKSQDTGLTPLEIQSQQQKLRQQQLQQQKFEAAASYLANATKLMQESNQDSHLSGTHNNNSSKNGNNNLMTMKA SISSPNTSVNSIQSPPSVNSVNGSGQGVSTGINVSGNNGRIEVGIGNSVNPYNGRIN*

**Sgf73 in Sus1-TAPspt7Δ**

MRSGDAEIKGIKPKVIEEYSLSQGSGPSNDSWKSLMSSAKDTPLQYDHMNRESLKKYFNPNAQLIEDPLDKPIQYRVCEKCGKPLALTAIVDHLENHCAGASGKSSTDPRDESTRETIRNGVESTGRNNNDDDNSNDNNNDDDDDDDNDD NEDDDDADDDDDNSNGANYKKNDSSFNPLKRSTSMESANTPNMDTKRSKT G**T**PQTFSSSI**K**KQKKVKQRNPTE**K**HLIDFNKQCGVELPEGGYCARSLTCK SHSMGAKRAVSGRSKPYDVLLADYHREHQTKIGAAAE**K**RAKQQELQKLQ**K**

QIQKEQKKHTQQQKQGQRSKQRNVNGGKSAKNGGKSTVHNGNNINEIGHV NLT**PEEETTQVLNGVSRSFPLPLESTVLSSVRYR**TKYFR**MREMFASSFSVKPGYTSPGYGAIHSRVGCLDLDRTTDYKFRVRTPQPI**NHLTNQNLNPKQIQRLQQQRALQAQLLSQQQQQQQQQQQHHSPQAQAQASTQQPTQGMVPNHFPGGATNSSFNANVSSKQIQQQQQQQQHKSQDTGLTPLEIQSQQQKLRQQQLQQQKFEAAASYLANATKLMQESNQDSHLSGTHNNNSSKNGNNNLMTMKA SISSPNTSVNSIQSPPSVNSVNGSGQGVSTGINVSGNNGRIEVGIGNSVNPYNGRIN*

Papai G, Frechard A, Kolesnikova O, Crucifix C, Schultz P, Ben-Shem A.Structure of SAGA and mechanism of TBP deposition on gene promoters.

Nature. 2020 Jan;577(7792):711-716. doi: 10.1038/s41586-020-1944-2. Epub 2020 Jan 22.PMID: 31969704

**Figure S2.** Levels of Ubp8 protein tagged with GFP in WT and *spt7*Δ mutant cells were analysed in three different experiments (1, 2 and 3) with two replicates of Ubp8-GFP*spt7*Δ in each (a and b) by western blotting of whole-cell extracts using an anti-GFP antibody. Levels of Pgk1 protein were also monitored and used as the loading control. Cropped blots are shown for clarity. Full-length blots are presented in Figure S6**.**

**
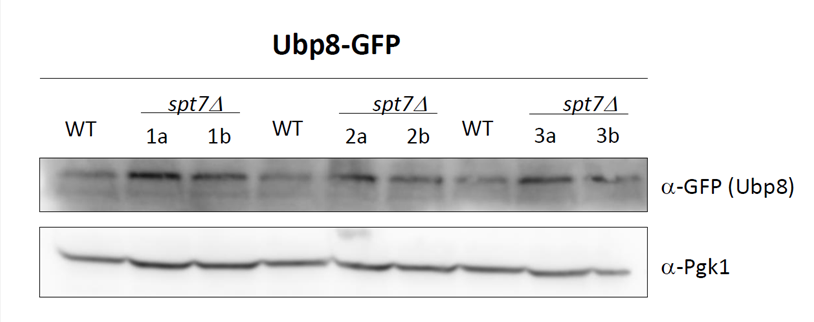
**

**Figure S3.** Full-length blot that was cropped for Fig. 1e

**
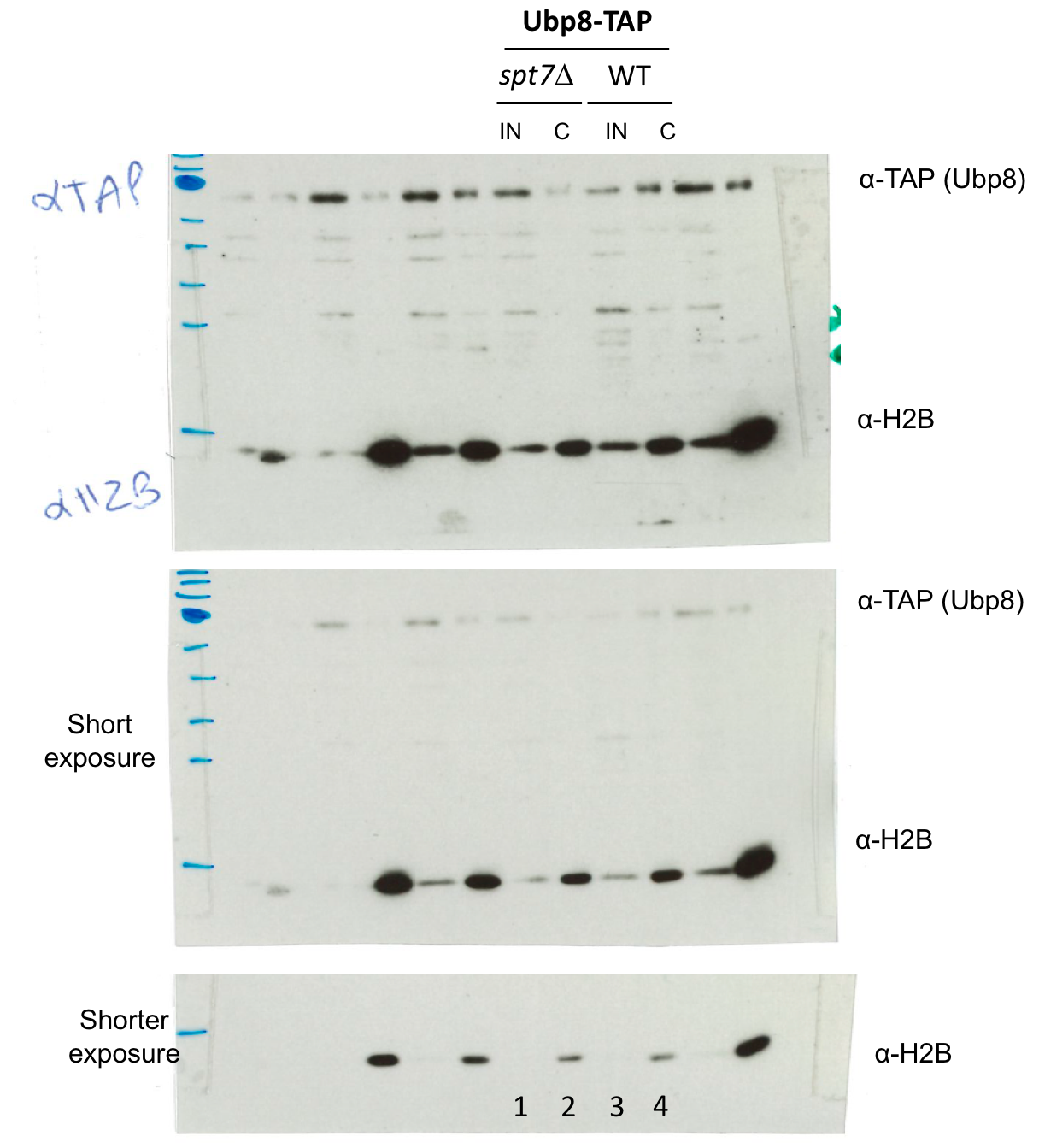
**

**Figure S4.** Full-length blot that was cropped for Fig. 4a.

**
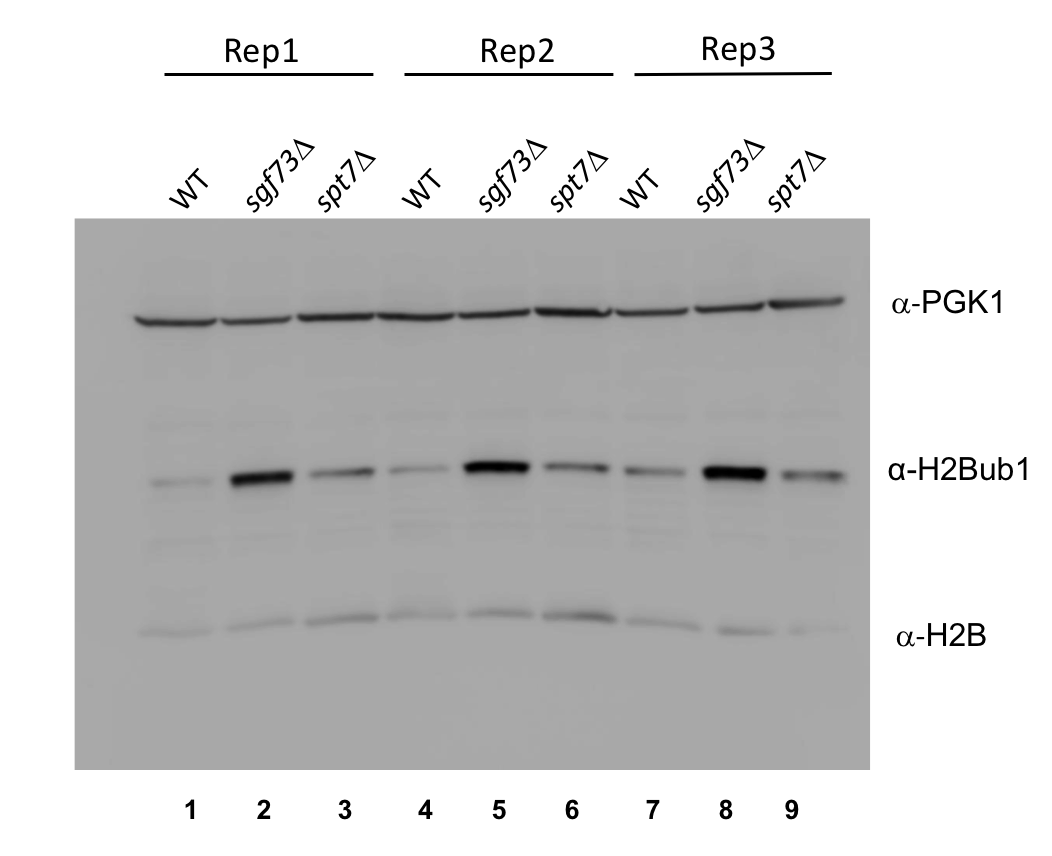
**

**Figure S5.** Full-length blot that was cropped for Fig. 4c (upper and lower panels).

**
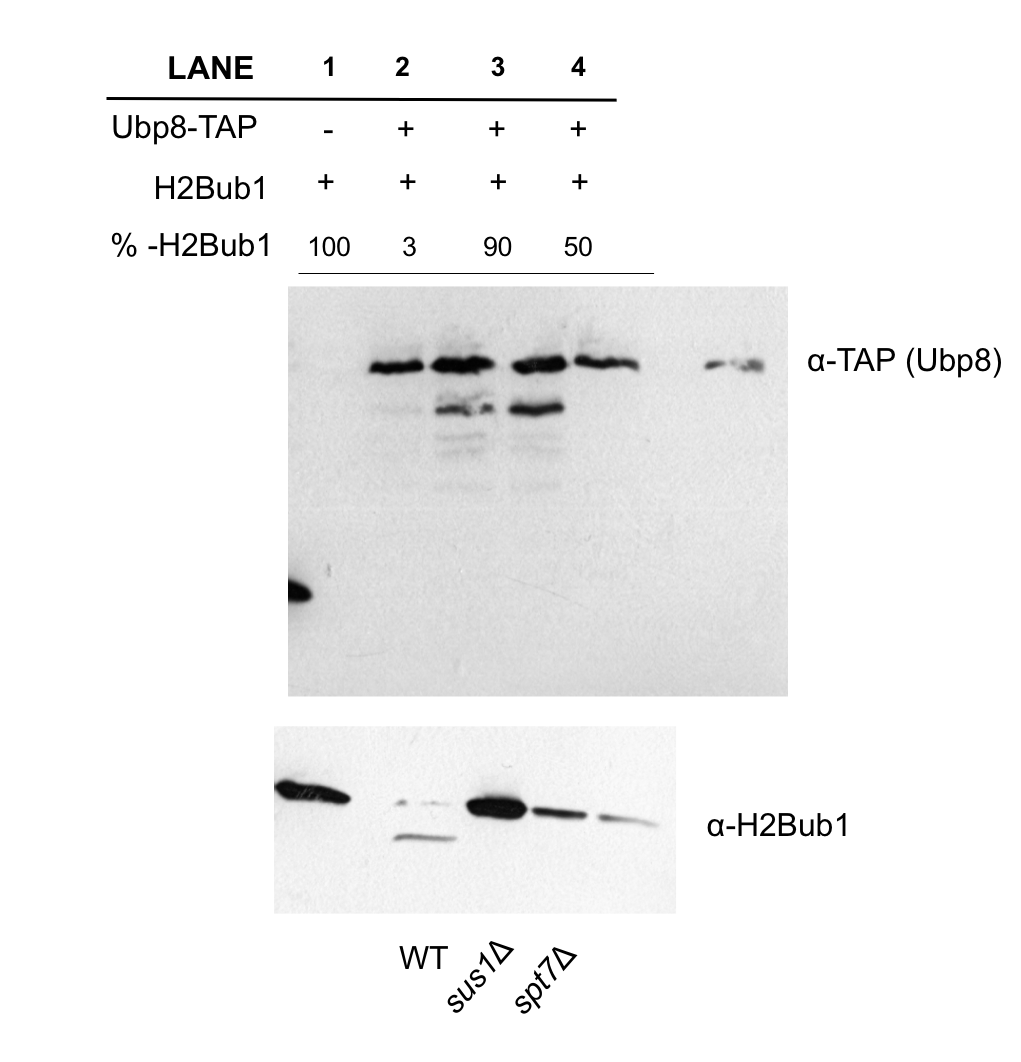
**

**Figure S6.** Full-length blots that were cropped for Figure S2

(upper panel)

**
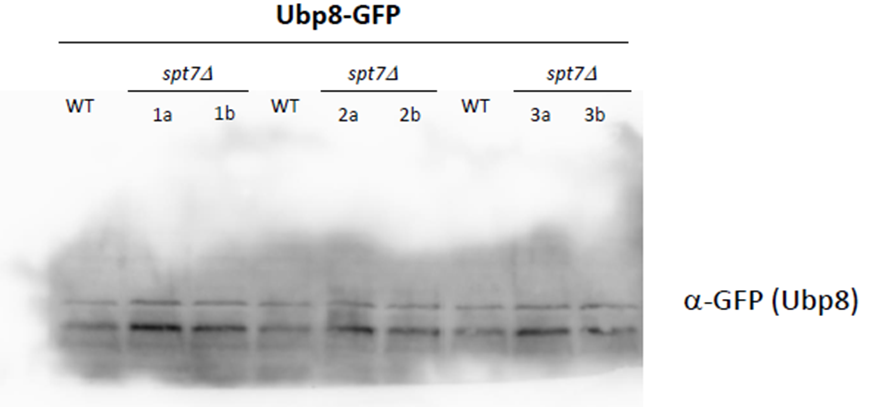
**

(lower panel)


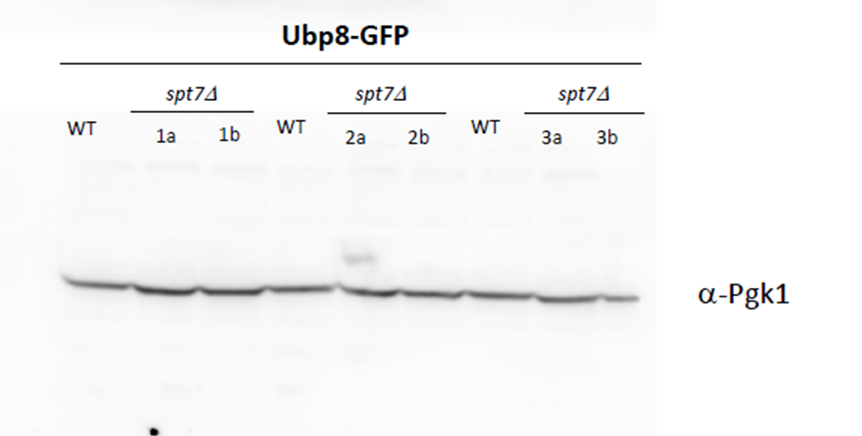


**Figure S7.** Different full microscope images used in Figure 2 and 3**.**

**Ubp8-GFP**

**GFP**

**
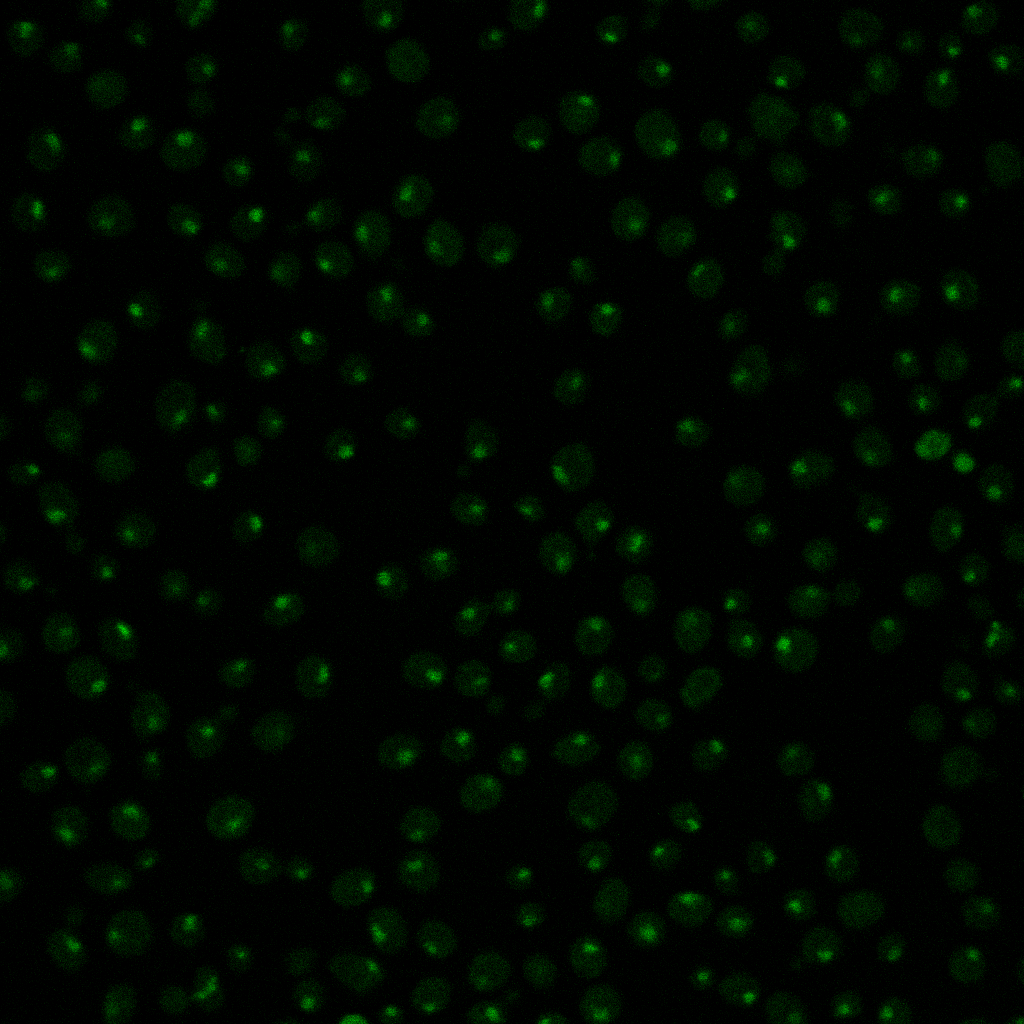
**

**DAPI**

**
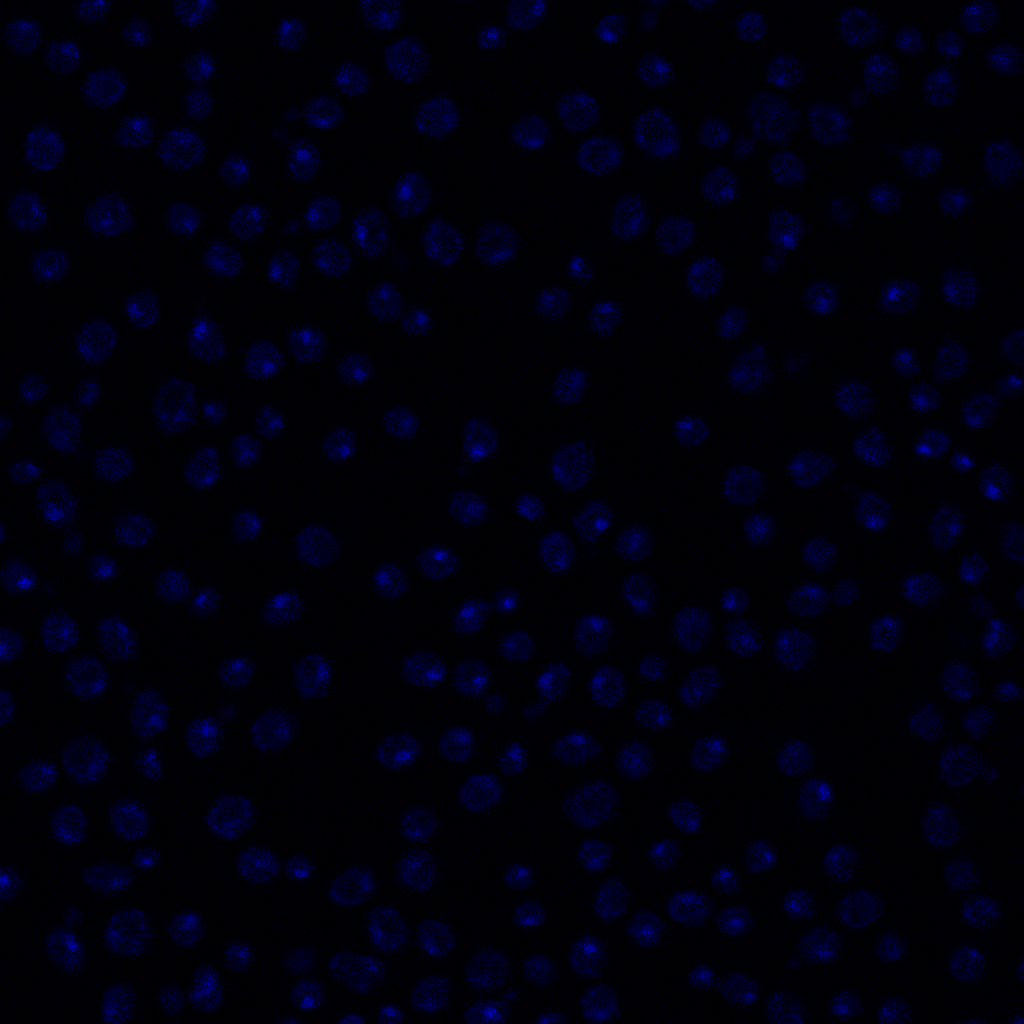
**

**Ubp8-GFPspt7Δ**

**GFP**

**
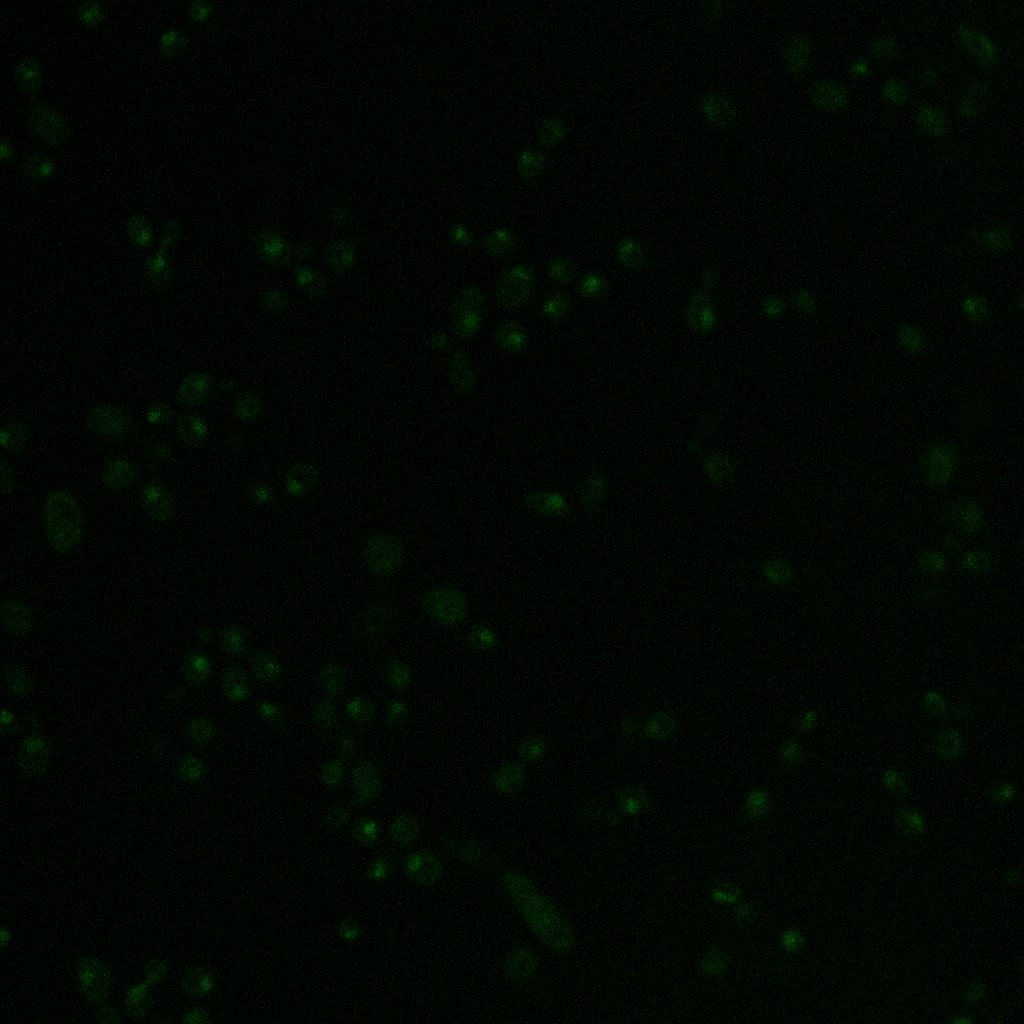
**

**Ubp8-GFPspt7Δ**

**DAPI**

**
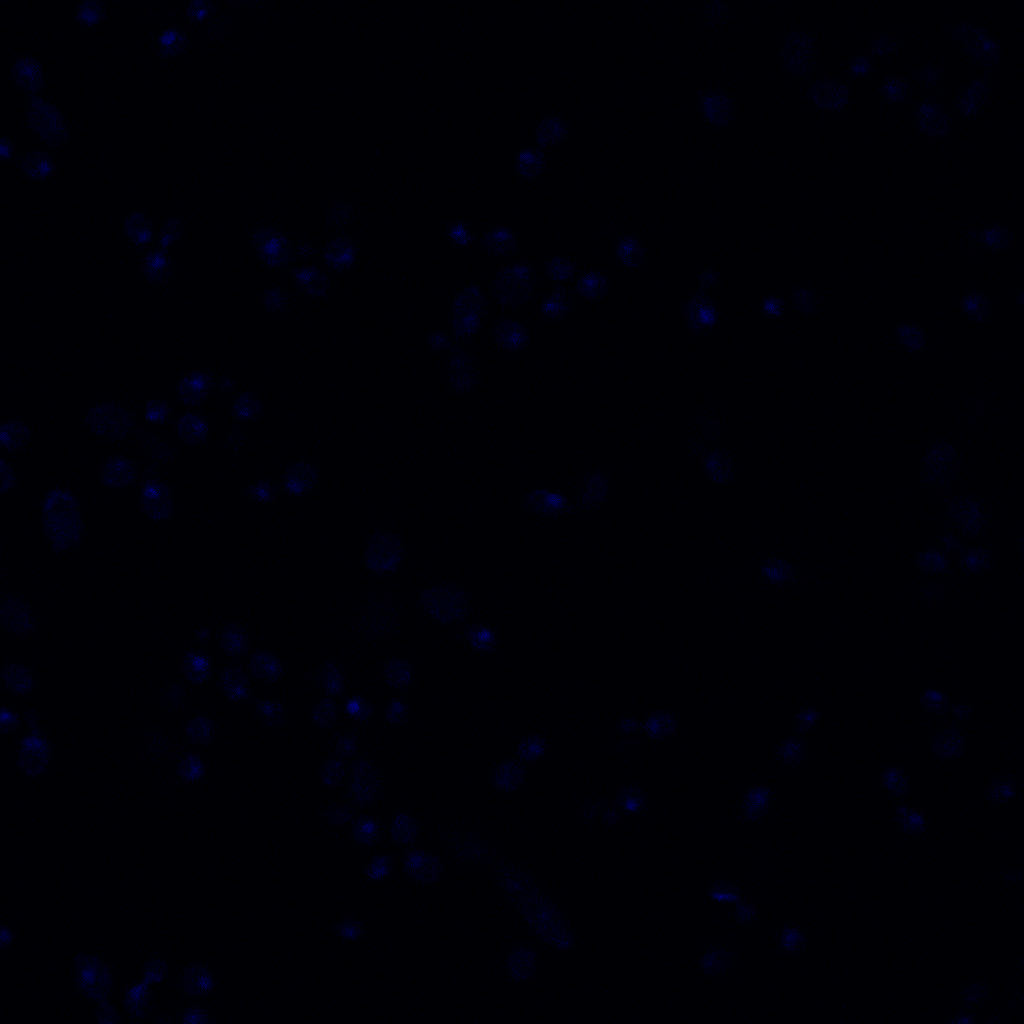
**

**Ubp8-GFPada1Δ**

**GFP**

**
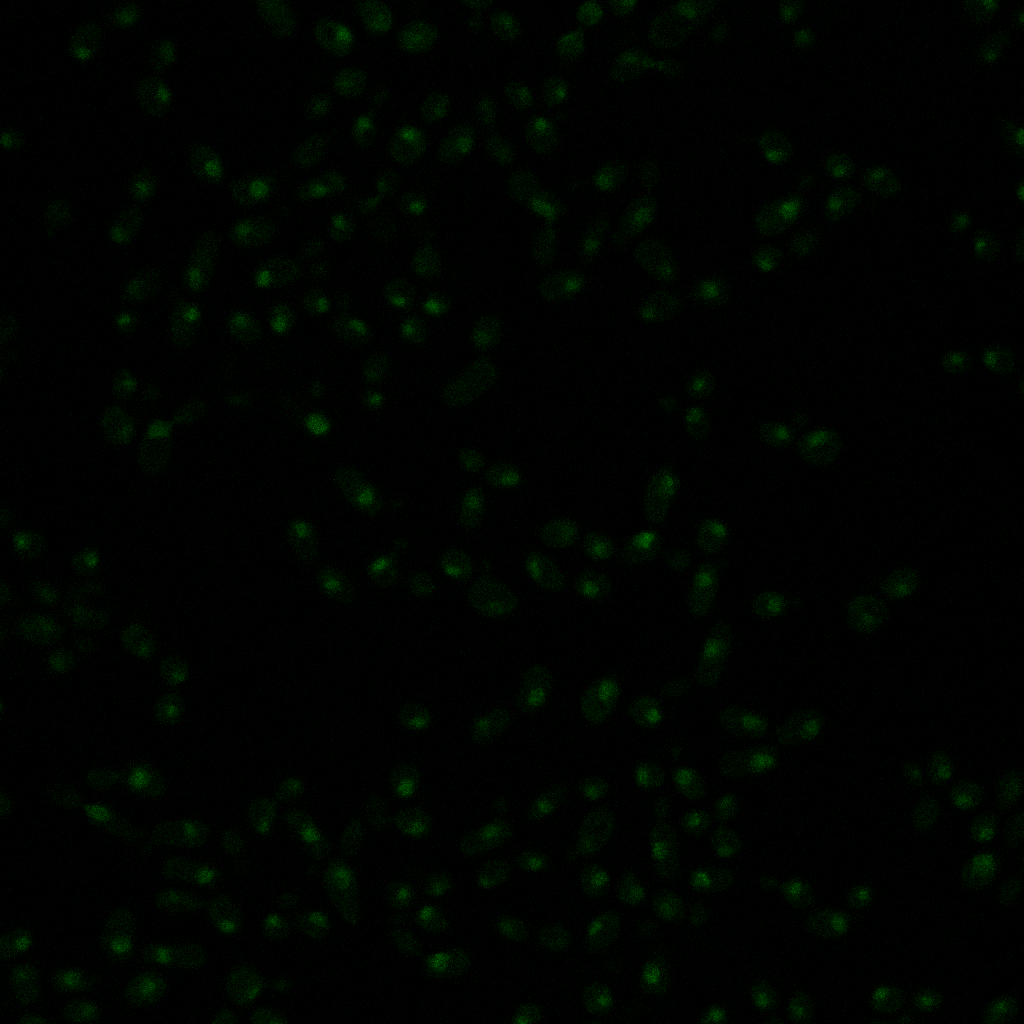
**

**Ubp8-GFPada1Δ**

**DAPI**

**
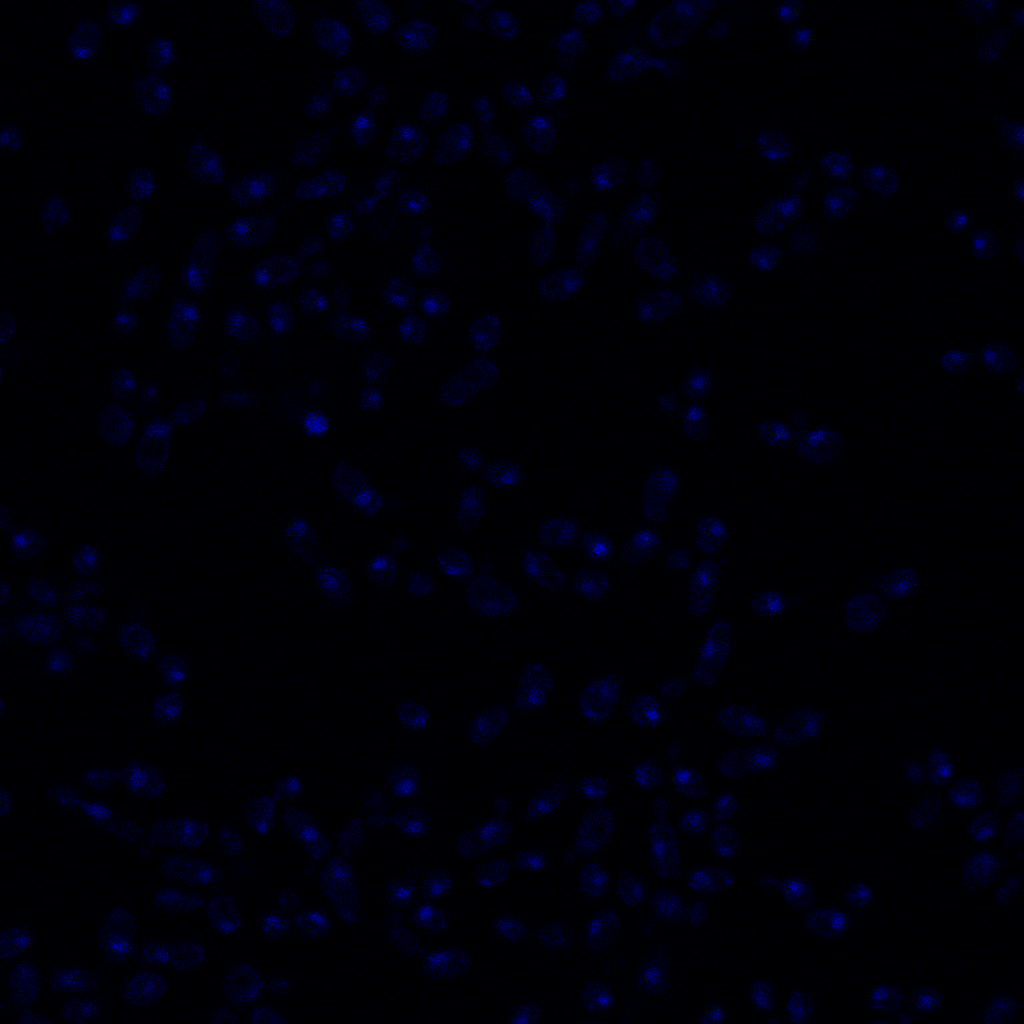
**

**Ubp8-GFPspt20Δ**

**GFP**

**
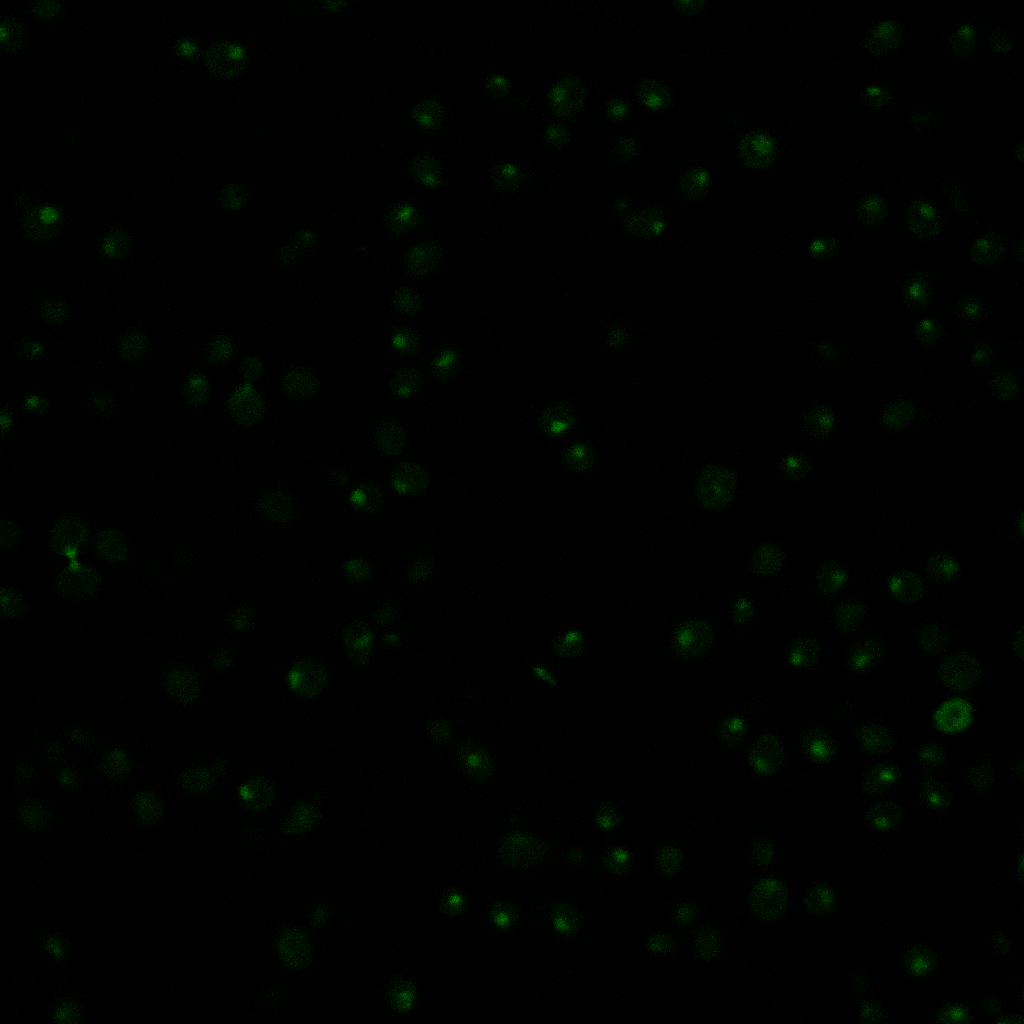
**

**Ubp8-GFPspt20Δ**

**DAPI**

**
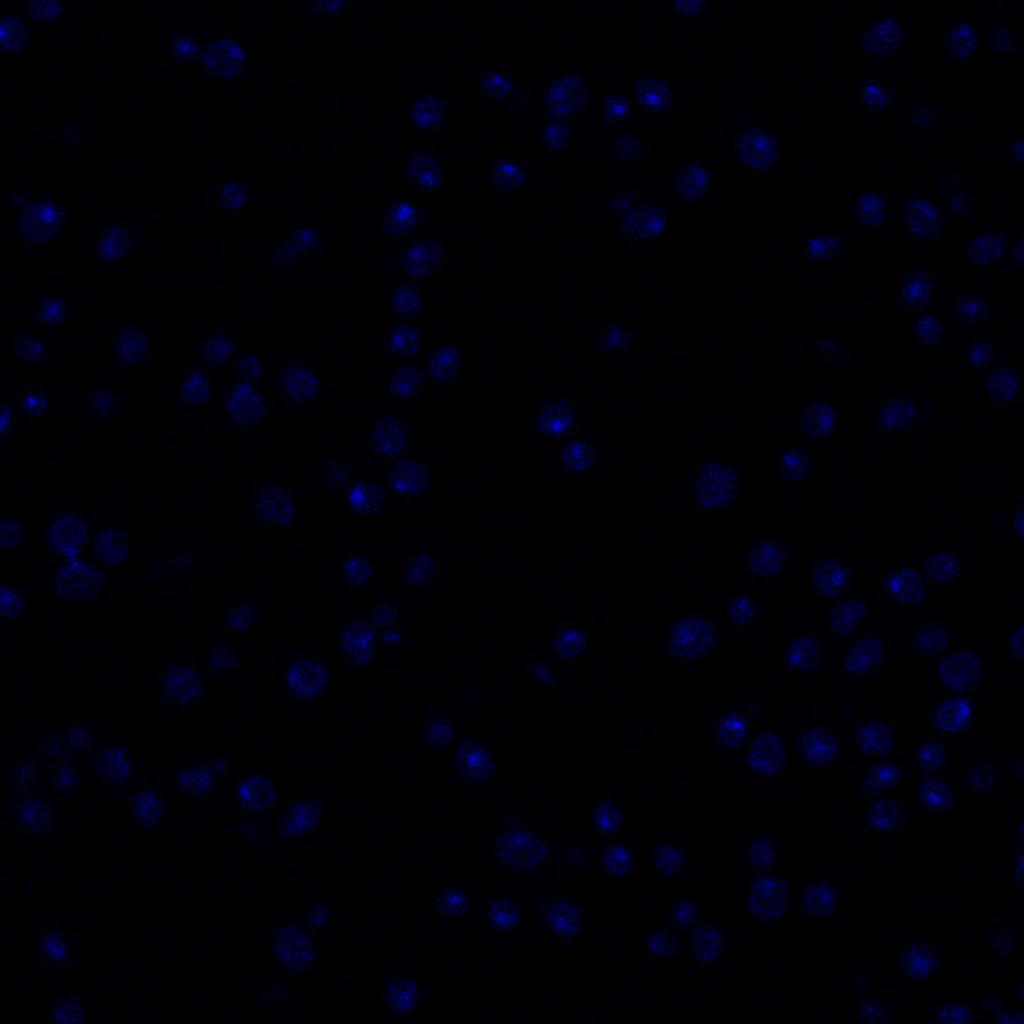
**

**Ubp8-GFPada1Δspt20Δ**

**GFP**

**
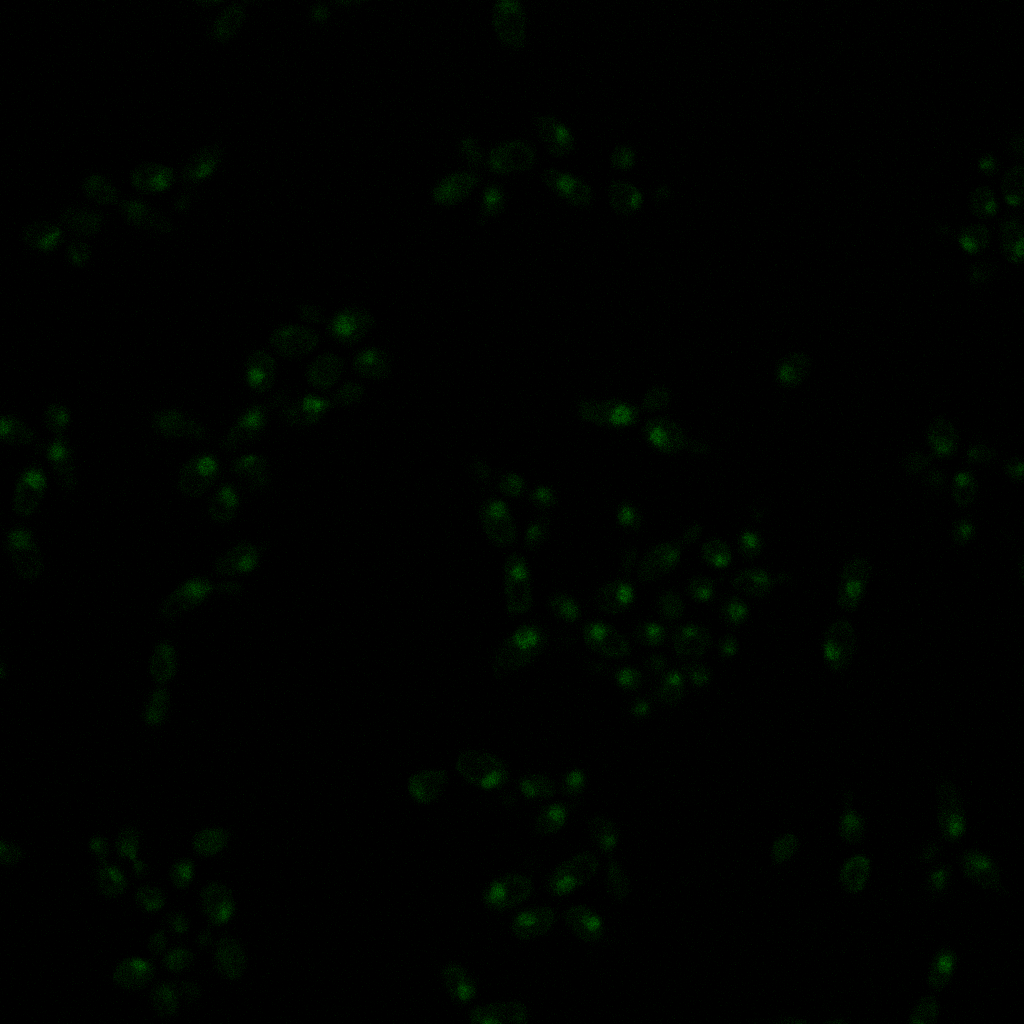
**

**Ubp8-GFPada1Δspt20Δ**

**DAPI**

**
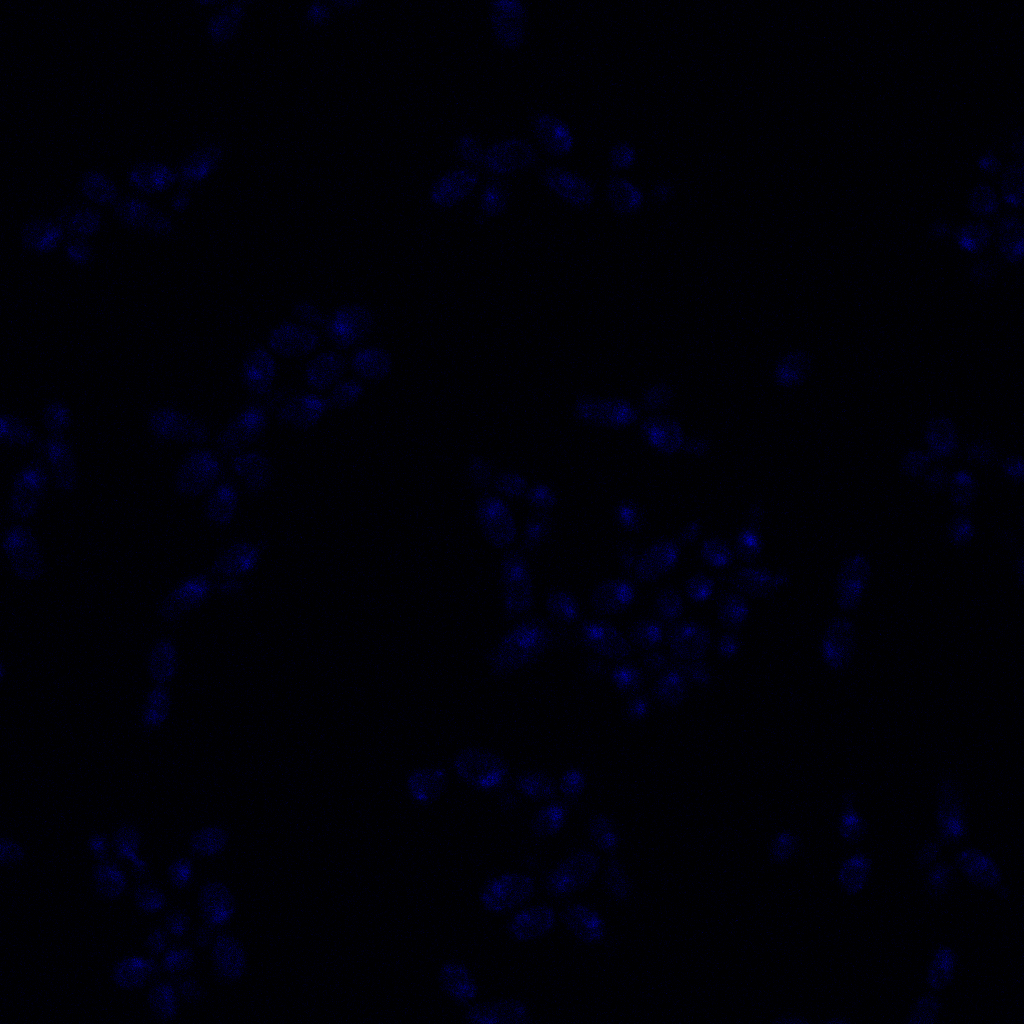
**

**Figure S8.** Detailed GFP Fluorescence microscopic quantification using IPython notebooks. To determine significant values between different experimental groups, the mean data were compared using one-way analysis of variance (ANOVA). Tukey's multiple comparisons test was also used. Values of *p<0.001 were considered significant.
